# Targeting the thioredoxin system as a novel strategy against B‐cell acute lymphoblastic leukemia

**DOI:** 10.1002/1878-0261.12476

**Published:** 2019-04-05

**Authors:** Klaudyna Fidyt, Agata Pastorczak, Agnieszka Goral, Kacper Szczygiel, Wojciech Fendler, Angelika Muchowicz, Marcin Adam Bartlomiejczyk, Joanna Madzio, Julia Cyran, Agnieszka Graczyk‐Jarzynka, Eugene Jansen, Elzbieta Patkowska, Ewa Lech‐Maranda, Deepali Pal, Helen Blair, Anna Burdzinska, Piotr Pedzisz, Eliza Glodkowska‐Mrowka, Urszula Demkow, Karolina Gawle‐Krawczyk, Michal Matysiak, Magdalena Winiarska, Przemyslaw Juszczynski, Wojciech Mlynarski, Olaf Heidenreich, Jakub Golab, Malgorzata Firczuk

**Affiliations:** ^1^ Department of Immunology Medical University of Warsaw Poland; ^2^ Postgraduate School of Molecular Medicine Medical University of Warsaw Poland; ^3^ Department of Pediatrics, Oncology, Hematology and Diabetology Medical University of Lodz Poland; ^4^ Department of Biostatistics and Translational Medicine Medical University of Lodz Poland; ^5^ Department of Radiation Oncology Dana‐Farber Cancer Institute Boston MA USA; ^6^ Centre for Health Protection National Institute for Public Health and the Environment Bilthoven The Netherlands; ^7^ Institute of Hematology and Transfusion Medicine Warsaw Poland; ^8^ Centre of Postgraduate Medical Education Warsaw Poland; ^9^ Newcastle Cancer Centre at the Northern Institute for Cancer Research Newcastle University Newcastle upon Tyne UK; ^10^ Department of Immunology, Transplantology and Internal Diseases Medical University of Warsaw Poland; ^11^ Department of Orthopaedics and Traumatology Medical University of Warsaw Poland; ^12^ Department of Laboratory Diagnostics and Clinical Immunology of Developmental Age Medical University of Warsaw Poland; ^13^ Department of Pediatrics, Hematology and Oncology Medical University of Warsaw Poland; ^14^ Centre for Preclinical Research and Technology Medical University of Warsaw Poland

**Keywords:** ALL, antioxidant enzymes, leukemia, oxidative stress, peroxiredoxin, thioredoxin

## Abstract

B‐cell precursor acute lymphoblastic leukemia (BCP‐ALL) is a genetically heterogeneous blood cancer characterized by abnormal expansion of immature B cells. Although intensive chemotherapy provides high cure rates in a majority of patients, subtypes harboring certain genetic lesions, such as *MLL* rearrangements or *BCR‐ABL1* fusion, remain clinically challenging, necessitating a search for other therapeutic approaches. Herein, we aimed to validate antioxidant enzymes of the thioredoxin system as potential therapeutic targets in BCP‐ALL. We observed oxidative stress along with aberrant expression of the enzymes associated with the activity of thioredoxin antioxidant system in BCP‐ALL cells. Moreover, we found that auranofin and adenanthin, inhibitors of the thioredoxin system antioxidant enzymes, effectively kill BCP‐ALL cell lines and pediatric and adult BCP‐ALL primary cells, including primary cells cocultured with bone marrow‐derived stem cells. Furthermore, auranofin delayed the progression of leukemia in *MLL*‐rearranged patient‐derived xenograft model and prolonged the survival of leukemic NSG mice. Our results unveil the thioredoxin system as a novel target for BCP‐ALL therapy, and indicate that further studies assessing the anticancer efficacy of combinations of thioredoxin system inhibitors with conventional anti‐BCP‐ALL drugs should be continued.

**Abbreviations**
ADE: adenanthin
ASK1: apoptosis signal‐regulating kinase 1
AUR: auranofin
BAP: biological antioxidant potential
BCP‐ALL: B‐cell precursor acute lymphoblastic leukemia
BCR‐ABL1: breakpoint cluster region‐Abelson kinase 1
BM‐MSC: bone marrow‐derived mesenchymal stem cells
CHOP: CCAAT/enhancer‐binding protein (C‐EBP) homologous protein
CLL: chronic lymphocytic leukemia
c‐MYC: cellular MYC
eIF2α: eukaryotic initiation factor 2 α
ER: endoplasmic reticulum
GRP78: glucose‐regulated protein 78
GSH: glutathione
H2A.X: histone 2A variant X
HO‐1: heme oxygenase‐1
MAPK: mitogen‐activated protein kinases
MLL: mixed lineage leukemia
MLLr: mixed lineage leukemia gene rearrangements
NRF2: nuclear factor‐like‐2
NSG: NOD.Cg‐Prkdc^scid^ Il2rg^tm1Wjl^/SzJ, The Jackson Laboratory
PARP: poly(ADP‐ribose) polymerase
PERK: protein kinase RNA‐like endoplasmic reticulum kinase
PRDX: peroxiredoxin
PTEN: phosphatase and tensin homolog
ROM: reactive oxygen metabolites
ROS: reactive oxygen species
TXNRD: thioredoxin reductase
TXN: thioredoxin
UPR: unfolded protein response

## 1 Introduction

B‐cell precursor acute lymphoblastic leukemia (BCP‐ALL) is an aggressive malignancy associated with abnormal expansion of immature B cells. The molecular background of BCP‐ALL is heterogeneous, and some known genetic aberrations that define genetic subtypes of the disease, including breakpoint cluster region‐Abelson kinase 1 (*BCR‐ABL1*) translocation, mixed lineage leukemia gene rearrangements (MLLr), hypodiploidy, and BCR‐ABL‐like subtype, are associated with poor prognosis (Hunger and Mullighan, 2015). Although the outcome of both childhood and adult BCP‐ALL has been substantially improved after the introduction of risk‐adapted therapy, treatment of high‐risk cytogenetic subtypes still remains a clinical challenge, for which novel therapeutic approaches are needed (O'Dwyer and Liesveld, 2017).

Targeting metabolic pathways aberrantly activated in malignant cells is recently emerging as a promising anticancer strategy. During tumorigenesis, activation of oncogenes, and increased metabolism and proliferation rate result in elevated production of reactive oxygen species (ROS), which contribute to redox imbalance and oxidative stress, one of the hallmarks of cancer cells (Glasauer and Chandel, 2014; Graczyk‐Jarzynka *et al*., 2017). To handle elevated ROS levels, cancer cells upregulate antioxidant enzymes and become reliant on these enzymes for their survival. Major cellular antioxidant machineries include glutathione (GSH) and thioredoxin (TXN) systems. TXN system is composed of several interdependent enzymes, such as thioredoxins (TXNs), thioredoxin reductases (TXNRDs), and peroxiredoxins (PRDXs) (Lu and Holmgren, 2014). TXN acts as a protein disulfide reductase. During substrate reduction, TXN gets oxidized and requires reactivation by NADPH‐dependent TXNRD. Among several known TXN substrates are peroxiredoxins 1–5, antioxidant enzymes which catalyze reduction of peroxides to water (Graczyk‐Jarzynka *et al*., 2017).

Redox imbalance and oxidative stress observed in cancer cells warrant targeting redox homeostasis in cancer therapy. However, the effects of ROS on cancer cells are two‐sided. Moderate, controlled ROS levels act as signaling molecules and contribute to cancer initiation and progression. On the other hand, when ROS levels exceed the cellular antioxidant capacity, they lead to macromolecule damage, cell cycle arrest, and cell death (Gorrini *et al*., 2013). Consequently, two opposite anticancer strategies interfering with ROS levels are possible, antioxidant and pro‐oxidant. The former, antioxidant approach, had been widely investigated, but the clinical benefits of these therapies in cancer patients are limited (Gorrini *et al*., 2013). The latter, pro‐oxidant approach, is based on increased basal ROS levels in cancer cells and the reliance of cancer cells on ROS removal by antioxidant enzymes (Glasauer and Chandel, 2014; Gorrini *et al*., 2013; Trachootham *et al*., 2009). Indeed, inhibitors of antioxidant enzymes of the TXN system selectively kill cancer cells (Graczyk‐Jarzynka *et al*., 2018; Klossowski *et al*., 2012; Liu *et al*., 2012; Stafford *et al*., 2018). The prominent examples in hematologic malignancies include a TXNRD1/2 inhibitor auranofin (AUR) and a PRDX1/2 and TXN1 inhibitor adenanthin (ADE). AUR is an approved, antirheumatic drug, which showed preclinical efficacy against B‐cell chronic lymphocytic leukemia (CLL) and has been already tested in phase 1/2 trial in CLL patients (NCT01419691). ADE induced differentiation of promyelocytic leukemia and demonstrated preclinical, antileukemic activity (Liu *et al*., 2012).

The inhibition of the TXN system leads to cancer cell death; however, the mechanisms and signaling pathways mediating the death are complex. Due to the crucial role of the TXN system in ROS removal, its blocking leads to elevation of ROS levels, oxidative damage, and cell death. Moreover, the anti‐apoptotic functions of some enzymes of the TXN system result from their direct interactions with the key signaling proteins. For instance, TXN1 interacts with the apoptosis signal‐regulating kinase 1 (ASK1) and prevents its pro‐apoptotic signaling. PRDXs are partners of a variety of oncogenes or tumor suppressors, such as mitogen‐activated protein kinases (MAPKs) (Kim *et al*., 2006), c‐MYC (Egler *et al*., 2005), or phosphatase and tensin homolog (PTEN) (Cao *et al*., 2009). Furthermore, in CLL cells, it was demonstrated that AUR triggered endoplasmic reticulum (ER) stress‐induced apoptosis (Fiskus *et al*., 2014). ER stress results from accumulation of unfolded or damaged proteins in the ER, which induces unfolded protein response (UPR) signaling pathways. Triggering UPR aims to alleviate the ER stress; however, sustained, unresolved ER stress ultimately leads to apoptosis. UPR signaling pathways are comprehensively described here (Moenner *et al*., 2007). As demonstrated recently, inhibition of cytosolic TXNRD1 disturbs folding of disulfides in the ER, establishing a direct link between cytosolic oxidative stress and the ER stress (Poet *et al*., 2017).

Although altered expression of some enzymes of the TXN system was already observed in selected hematologic malignancies (Agrawal‐Singh *et al*., 2012; Sewastianik *et al*., 2016; Trzeciecka *et al*., 2015), our knowledge on the influence of these enzymes on cancer progression and response to therapy of BCP‐ALL is currently limited. In this study, we aimed to validate antioxidant enzymes of the TXN system as putative therapeutic targets in BCP‐ALL. We provide data on the expression of the TXN system antioxidant enzymes in BCP‐ALL cell lines and primary cells and evaluate the antileukemic efficacy of the inhibitors of these enzymes *in vitro* and *in vivo*. Furthermore, we demonstrate that in BCP‐ALL cells AUR and ADE induce oxidative stress as well as ER stress, which finally result in apoptotic cell death.

## 2 Materials and methods

### 2.1 Cell culture

Human BCP‐ALL cell lines (697, REH) and B‐cell lymphoma cell line RL were purchased from ATCC (Manassas, VA, USA) and BCP‐ALL cell lines (SEM, SD1, BV173, SUP‐B15) from DSMZ (Braunschweig, Germany). These cell lines represent subtypes of BCP‐ALL harboring various genetic alterations, such as *MLL‐AF4* (SEM), *BCR‐ABL1* (BV173, SUP‐B15, SD1), *E2A‐PBX1* (697), and *TEL‐AML1* (REH). For mechanistic studies, we selected two cell lines representing the genetic subtypes with poor prognosis: SEM and BV173, and in selected experiments also primary BCP‐ALL blasts or their primografts. All cell lines were maintained in RPMI 1640 medium (Gibco, Paisley, UK) supplemented with 10% FBS (HyClone, Logan, UT, USA) and 1% penicillin/streptomycin solution (Sigma‐Aldrich, St. Louis, MO, USA) in a humidified atmosphere at 37 °C and 5% CO_2_. The cells were routinely checked for Mycoplasma contamination.

### 2.2 Chemicals

Adenanthin (Faces Biochemical Co., Wuhan, China) and auranofin (Santa Cruz Biotechnology, Dallas, TX, USA, and Sigma‐Aldrich) were dissolved in DMSO at 10 mm concentration. All drugs were aliquoted and stored at −20 °C. In all assays, control groups were treated with DMSO (Sigma‐Aldrich).

### 2.3 Leukemic patients

#### 2.3.1 Pediatric BCP‐ALL patients

In total, *n* = 129 childhood BCP‐ALL patients of specified cytogenetic or/and molecular ALL subtypes were enrolled into gene expression analysis. Patients were diagnosed and treated according to ALL IC BFM 2009 protocol in the clinical centers of the Polish Pediatric Leukemia/Lymphoma Study Group between August 2014 and December 2016. The research protocol was approved by the Bioethics Committee of the Medical University of Lodz, and informed written consent was obtained from all participants and/or their parents. Mononuclear leukocytes were extracted from bone marrow taken for routine diagnostic procedures and processed within 24 h. All bone marrow aspirates had more than 80% blasts as confirmed by microscopic and flow cytometry analyses. Detailed characteristics of pediatric patients enrolled into gene expression analysis are listed in Table S3.

#### 2.3.2 Adult BCP‐ALL patients

Adult patients (*n* = 12) were enrolled at the Institute of Hematology and Transfusion Medicine in Warsaw after obtaining appropriate informed written consent under the approval of the Bioethics Committee of Medical University of Warsaw. Treatment and risk stratification of the patients were carried out according to PALG ALL6 protocol. The collected bone marrow was processed within 24 h. The percentage of blasts in the isolates was confirmed using flow cytometry. The samples that contained < 80% blasts were purified using EasySep™ Human CD19 Positive Selection Kit (STEMCELL Technologies, Vancouver, BC, Canada). Detailed characteristics of adult patients enrolled into gene expression analysis are listed in Table S4. The study methodologies conformed to the standards set by the Declaration of Helsinki.

### 2.4 Isolation of BCP‐ALL patients’ serum

Blood samples were collected from pediatric BCP‐ALL patients from Pediatric Hospital of Medical University of Warsaw [*n* = 35, median age = 5.57 (min–max: 1.42–16.58)] and adult BCP‐ALL patients obtained from Institute of Hematology and Transfusion Medicine in Warsaw [*n* = 17, median age = 47 (min–max: 20–72)] under the approval of the Bioethics Committee of Medical University of Warsaw. Control groups consisted of age‐related 21 pediatric and 14 adult healthy donors. Following blood collection, the samples were stored at room temperature for 1–4 h to form a clot and then centrifuged at 1000 ***g*** for 10 min. Serum‐containing supernatants were collected and stored at −80 °C.

### 2.5 Isolation of normal CD19^+^ and CD34^+^ cells

Normal CD19^+^ and CD34^+^ cells were isolated from healthy donors’ peripheral blood obtained from Regional Blood and Hemotherapy Center in Warsaw. Normal peripheral blood mononuclear cells (normal PBMC) were isolated using density gradient medium – Lymphoprep™ (1.077 g·mL^−1^; Axis‐Shield, Oslo, Norway). Subsequently, CD19^+^ cells were isolated with EasySep™ Human CD19 Positive Selection Kit (STEMCELL Technologies), and CD34^+^ cells with EasySep™ Human CD34 Positive Selection Kit (STEMCELL Technologies). Germinal center B cells (GC B cells) were isolated as described previously (Trzeciecka *et al*., 2015).

### 2.6 Analysis of redox biomarkers in sera samples

Markers of redox homeostasis in the serum of pediatric and adult BCP‐ALL patients were determined by the following assays (Diacron, Grosseto, Italy): reactive oxygen metabolites (ROM, which quantifies hydroperoxides by the Fenton reaction), biological antioxidant potential (BAP, which measures the concentration of antioxidants as agents that can reduce iron from the ferric to ferrous form), and protein thiol levels (SHP, which measures all free thiol groups in proteins), using DxC auto‐analyzer (Beckman Coulter, Woerden, the Netherlands) as previously described (Jansen *et al*., 2015). These three assays gave stable results, also after storage of serum samples at −80 °C for longer periods (Jansen *et al*., 2015). In the analysis, we included 17 adult and 35 pediatric BCP‐ALL sera samples. As controls, we used sera collected from age‐matched healthy subjects (21 pediatric and 14 adult).

### 2.7 Assessment of mRNA levels by qPCR in BCP‐ALL cell lines and normal CD19^+^ and CD34^+^ cells

For detection of *PRDX1*,* TXN1*, and *TXNRD1* mRNA levels, BCP‐ALL cell lines were seeded onto six‐well plates at 0.2 × 10^6^ cells·mL^−1^ density and cultured for 48 h. To evaluate the *GRP78* and *CHOP* mRNA level, SEM cells were seeded onto six‐well plates at 0.2 × 10^6^ cells·mL^−1^ density and treated with AUR and ADE for 3, 6, and 24 h. Before RNA isolation, cells were washed with phosphate‐buffered saline (PBS), pelleted, and suspended in 0.5 mL of TRIzol reagent (Roche, Mannheim, Germany). Normal CD19^+^ and CD34^+^ cells were suspended in TRIzol reagent directly after isolation by magnetic beads (EasySep™ positive selection kits).

The RNA was isolated according to the manufacturer's protocol. The purity and concentration of isolated RNA was measured by NanoDrop 2000c spectrophotometer (Thermo Fisher Scientific, Waltham, MA, USA). Hematopoietic Progenitor Cell (CD34^+^) pooled total RNA isolated from multiple donors was also purchased from MACS (Miltenyi, Bergisch Gladbach, Germany; cat. no. 130‐093‐167). Subsequently, 0.1–0.5 μg of RNA was incubated with DNase (Sigma‐Aldrich) and used for cDNA synthesis with the avian myeloblastosis virus (AMV) reverse transcriptase (EURx, Gdansk, Poland) and Transcriptor First Strand cDNA Synthesis Kit (Roche) for cell lines and normal cells, respectively. Assessment of the expression of *PRDX1*,* TXN1*,* TXNRD1, GRP78*,* CHOP*, and *RPL29* was evaluated as described previously (Muchowicz *et al*., 2015). The primers’ sequences are listed in Table S5.

### 2.8 Assessment of mRNA levels by qPCR in primary BCP‐ALL samples

RNA from BCP‐ALL pediatric patients was isolated according to the manufacturer's protocol (Ambion by Life Technologies, Carlsbad, CA, USA) with additional step of DNase digestion with RNase‐Free DNase Set (Qiagen, Hilden, Germany). The concentration and quality of isolates were determined by ultraviolet spectrophotometry (NanoDrop 8000; Thermo Fisher Scientific, Waltham, MA, USA). RNA from BCP‐ALL adult patients was isolated and incubated with DNase as described for BCP‐ALL cell lines. A total of 1 μg of RNA was used for cDNA synthesis using the High‐Capacity cDNA Reverse Transcription Kit (Thermo Fisher Scientific, Waltham, MA, USA) and Transcriptor First Strand cDNA Synthesis Kit (Roche) for pediatric and adult patients, respectively. Then, 30 ng of cDNA was used for each qPCR. The expression of *PRDX1*,* TXN1*,* TXNRD1* target genes, and *RPL29* (ribosomal protein L29) as a reference gene was measured in duplicates by a fluorescence‐based kinetic qPCR. The reaction was performed using Mx3000P qPCR System (Agilent Technologies, Santa Clara, CA, USA) in combination with the intercalating fluorescent dye Fast SYBR Green Master Mix (Thermo Fisher Scientific, Waltham, MA, USA) in accordance with the manufacturer's instructions. Detailed patients’ characteristics are presented in Tables S3 and S4. Sequences of primers designed for qPCR are listed in Table S5. For the analysis of qPCR data, the relative gene expressions were calculated using cycle threshold (*C*_t_) values and are presented as $2^{-\Delta C_{t}}$.

### 2.9 Immunoblotting

For immunoblotting, cells were lysed in a lysis buffer (10% glycerol, 1.0% Triton X‐100, 150 mm NaCl, 5 mm EDTA, 50 mm Hepes, pH 7.4) containing Complete Protease Inhibitor Cocktail (Roche Diagnostics) and Complete Phosphatase Inhibitor Cocktail (Roche Diagnostics). Total protein concentration was accessed using Pierce BCA Protein Assay Kit (Thermo Fisher Scientific, Rockford, IL, USA), and subsequently 7 μg of whole‐cell protein lysates was separated in 10–15% polyacrylamide gels. Following SDS/PAGE, proteins were transferred to Protran nitrocellulose membranes (Schleicher and Schuell BioScience, Dassel, Germany), blocked in 5% nonfat dry milk or BSA, and incubated with following primary antibodies: anti‐PRDX1, anti‐TXN1, anti‐TXNRD1, anti‐HO‐1, anti‐GRP78, anti‐p‐eIF2α, anti‐eIF2α, anti‐PARP, and anti‐α‐tubulin, followed by the incubation with HRP‐linked secondary antibodies. More detailed information about primary antibodies is listed in Table S6. For signal development, SuperSignal Chemiluminescent Substrate (Thermo Fisher Scientific, Rockford, USA) and ChemiDoc Imaging System (Bio‐Rad Laboratories, Hercules, CA, USA) were used. Densitometric quantifications were performed using image lab Software (Bio‐Rad Laboratories).

### 2.10 MTT assay

To assess cytostatic/cytotoxic effects of AUR and ADE, we used 3‐(4,5‐dimethylthiazol‐2‐yl)‐2,5‐diphenyltetrazolium bromide (MTT) assay.

*BCP‐ALL cell lines* were cultured in their medium as described above, seeded onto 96‐well plates at 0.2 × 10^6^ cells·mL^−1^ density, and treated with drugs at particular dose range (0.0625–2 μm for AUR and 0.125–4 μm for ADE) in a total volume of 150 μL, 4 replicates per each concentration. As a background control, cells were exposed to 1% SDS. After 48 h, 5 mg·mL^−1^ MTT solution (Sigma‐Aldrich) was added to each well, followed by 2‐ to 4‐h incubation, depending on a cell line. In order to dissolve formazan crystals, solubilization buffer (10% SDS, 0.01 m HCl) was added, and the next day, absorbance was measured at 560 nm using a microplate reader (ASYS UVM 340; Biochrom, Berlin, Germany).

*Primary leukemic blasts* were suspended at a final concentration of 1–2 × 10^6^ cells·mL^−1^ in culture medium containing: RPMI 1640 (Sigma‐Aldrich), 20% FBS (Gibco, Thermo Fisher, Waltham, MA, USA), 2 mm l‐glutamine, 200 mg·mL^−1^ of gentamycin, and ITS (5 mg·mL^−1^ of insulin, 5 mg·mL^−1^ of transferrin, and 5 ng·mL^−1^ of sodium selenite). The 10 mm stocks of ADE and AUR were further diluted in RPMI 1640 in serial twofold dilutions, to obtain 0.0156–2 μm and 0.25–8 μm concentration ranges for AUR and ADE, respectively. Cell suspension (80 μL) was dispensed into 96‐well microculture plates containing 20 μL of the drug dilutions. Each drug concentration was tested in 2–3 replicates. Positive control (cells with vehicle alone) and blanks (medium alone) were included. Plates were incubated in humidified incubator in 5% CO_2_ at 37 °C. After 92 h, 10 μL MTT solution (5 mg·mL^−1^ MTT in saline stored at −20 °C) was added, and after shaking for 5 min, plates were incubated for 6 h at cell culture incubator. After incubation, formazan crystals were dissolved with 100 μL of 0.04 N HCl/isopropyl alcohol (acid isopropanol). The optical density (OD) was measured with a microplate reader Halo MPR‐96 (Dynamica Scientific, Newport Pagnell, UK) with 550‐nm filter. After the background subtraction, OD of each sample was normalized to untreated controls (DMSO). Viability of the cells is presented as percentage of the controls.

*Normal peripheral blood mononuclear cells (normal PBMC)* were isolated from peripheral blood of healthy donors using density gradient medium – Lymphoprep™ (1.077 g·mL^−1^; Axis‐Shield). Cells were seeded onto 96‐well plates at 1.0 × 10^6^ cells·mL^−1^ density and treated with drugs at a dose range of 0.125–2 μm for AUR and 0.25–2 μm for ADE in a total volume of 100 μL, 4 repeats per each concentration, for 4 days. The latter steps of the assay were performed as described for BCP‐ALL cell lines.

### 2.11 ROS assessment

Assessment of ROS levels was performed using CM‐H_2_‐DCFDA dye (Molecular Probes, Eugene, OR, USA) according to the manufacturer's protocol. Briefly, cells were seeded at 0.2 × 10^6^ cells·mL^−1^ density and stained with the dye for 30 minutes at 37 °C, using 5 μm CM‐H_2_‐DCFDA for 1‐, 2‐, 4‐, and 6‐h treatment with AUR or ADE (SEM, BV173), or 10 μm CM‐H_2_‐DCFDA for 6‐h treatment (BV173). The green fluorescence intensity of all live cells (gated using FSC and SSC parameters) was assessed by flow cytometry using Canto II (BD Biosciences, Franklin Lakes, NJ, USA).

### 2.12 Assessment of dead cells with propidium iodide staining

SEM and BV173 cells were seeded onto 96‐well plate at a density 0.2 × 10^6^ cells·mL^−1^ and pretreated with 1 mm of pyruvate for 30 min. Subsequently, cells were treated with indicated concentrations of AUR or ADE for 24 h and stained with propidium iodide (PI) at final concentration of 1 μg·mL^−1^ (Sigma‐Aldrich). PI‐positive cells were accessed by flow cytometry using Canto II (BD Biosciences).

### 2.13 Assessment of dead cells with 7AAD staining

Normal human PBMC and primograft BCP‐ALL cells were isolated as described above. Both normal and malignant cells were seeded onto 12‐well plates at a density of 1 × 10^6^ cells·mL^−1^ and treated with 0.5 and 1.0 μm of AUR for 4 days in monoculture. Following the treatment, PBMC were stained with anti‐hCD19 and anti‐hCD3 and primografts with anti‐hCD19 only. Prior to acquisition, normal and malignant cells were stained with 7AAD (BioLegend, San Diego, CA, USA) and analyzed by flow cytometry using Canto II (BD Biosciences).

### 2.14 BM‐MSC isolation and culture

Primary bone marrow‐derived mesenchymal stem cells (BM‐MSC) were obtained from bone marrow of two adult individuals (aged between 20 and 42 years) who underwent hip replacement surgery and were isolated according to the protocol described in Pal *et al*. (2016). Prior to sample collection, an appropriate individual written consent was obtained and an approval was given by Bioethical Committee of Medical University of Warsaw and the Newcastle and North Tyneside 1 Research Ethics Committee. Isolated BM‐MSC were cultured according to the protocol described in Pal *et al*. (2016). The identity and quality of the BM‐MSC was confirmed by the assessment of the positive (CD73, CD105, CD90) and negative (CD45, CD34, CD19) markers by flow cytometry as well as by their potential to differentiate into adipocytes, osteoblasts, and chondroblasts. Validation of isolated BM‐MSC is presented in Fig. S1. Antibodies used for flow cytometry are listed in Table S6.

### 2.15 Primograft generation

Animal experiments were performed under the approval given by Ethics Committee of the Medical University of Warsaw. NOD.Cg‐Prkdc^scid^ Il2rg^tm1Wjl^/SzJ, The Jackson Laboratory (NSG), mice were used for BCP‐ALL blasts’ propagation. For this purpose, cryopreserved primary ALL samples were thawed and 2–5 × 10^6^ cells were transplanted intrafemorally or via tail vein injections. The whole blood staining was performed to evaluate the engraftment of leukemic cells. Accordingly, cells were stained with anti‐mCD45, anti‐hCD45, anti‐hCD19 antibodies (Table S6) and analyzed by flow cytometry using Canto II (BD Biosciences). The mice were humanely killed when percentage of human blasts (hCD45^+^hCD19^+^/mCD45^+^ × 100%) in peripheral blood reached around 100% and was accompanied by visible signs of illness, such as inactivity and splenomegaly. Primograft ALL cells were collected from spleens and bone marrows of leukemic mice. All primografts used in *ex vivo* analysis contained > 80% CD19.

### 2.16 Drug treatment in the BCP‐ALL‐MSC coculture model

For testing drug cytotoxic/cytostatic effects, BCP‐ALL primografts were cocultured with BM‐MSC (Pal *et al*., 2016). To this end, BM‐MSC were seeded onto 48‐well plate at a density of 2.5–5.0 × 10^4^ cells per well, in DMEM (low glucose) supplemented with 20% FBS (Gibco), 2 mm of l‐glutamine (Sigma‐Aldrich), and 1% penicillin/streptomycin solution (Sigma‐Aldrich). The next day, BCP‐ALL primograft cells were added at a density of 0.5 × 10^6^ cells·mL^−1^ in SFEM II media (STEMCELL Technologies) supplemented with 20% FBS (Gibco), 20 ng·mL^−1^ of recombinant IL‐3 (R&D Systems, Minneapolis, MN, USA), 10 ng·mL^−1^ of recombinant IL‐7 (R&D Systems), and penicillin/streptomycin solution (Sigma‐Aldrich). After 24 h of coculture, cells were treated with auranofin (AUR) or adenanthin (ADE) at indicated concentration ranges, in a final volume of 700 μL. For each drug concentration, samples were run in duplicate. After 5 days, cells in suspension (BCP‐ALL cells) and adherent cells (BCP‐ALL+MSC) were collected and the number of viable cells was assessed in a hemocytometer. The numbers of viable BCP‐ALL cells in control groups after 5‐day‐long coculture exceeded the number of seeded cells, confirming the growth‐supporting role of BM‐MSC. Primograft samples used in drug‐testing experiments are listed in Table S7.

### 2.17 *In vivo* experiments

All experiments involving mice were carried out according to the EU Directive 2010/63/EU for animal experiments and the Polish legislation on animal experiments of the Polish Ministry of Science and Higher Education from February 26, 2015. Mice were maintained in SPF animal facility equipped with individually ventilated cages. In planning, design, execution, and presenting of animal experiments, we followed the Animal Research: Reporting of *In Vivo* Experiments (ARRIVE) guidelines for reporting animal research (Kilkenny *et al*., 2010). All animal experiments were performed under the approval of the Ethics Committee of the Medical University of Warsaw.

Cryopreserved primograft samples were thawed, and 0.5 × 10^6^ cells were transplanted through tail vein injections to 8‐ to 12‐week‐old male and female NSG mice (NOD.Cg‐Prkdc^scid^ Il2rg^tm1Wjl^/SzJ, The Jackson Laboratory). The engraftment of the human cells was evaluated in the whole blood collected from cheek vein, once a week, via staining with anti‐mCD45, anti‐hCD45, anti‐hCD19 antibodies (Table S6). The percentage of human blasts was determined using the formula: (hCD45^+^hCD19^+^/mCD45^+^) × 100%. When the percentage of the human cells exceeded 1% murine mCD45^+^ cells, randomly selected mice were treated with 10 mg·kg^−1^ of auranofin or DMSO (control) through intraperitoneal injections, once a day for 3 weeks (5 days of treatment/2 days of break schedule). The leukemia progression was monitored once a week by whole blood staining, as described above. The mice were euthanized by cervical dislocation when visible signs of sickness were observed, such as splenomegaly or loss in hind paw reflex, in parallel with five times higher ratio of human blasts to murine leukocytes in the blood in two consecutive measures. No effect of sex on the development of leukemia or on the effectiveness of therapy was observed.

### 2.18 Detection of DNA oxidative damage

SEM cells were seeded onto 24‐well plate in RPMI medium supplemented with 10% FBS and penicillin/streptomycin solution at 0.2 × 10^6^ cells·mL^−1^ density and subjected to AUR or ADE treatment for 24 h. BCP‐ALL primografts were seeded onto 48‐well plate at a density of 1 × 10^6^ cells·mL^−1^ in SFEM II medium supplemented as described above. Induction of DNA oxidative damage was accessed by Muse^®^ H2A.X Activation Dual Detection Kit (Merck Millipore, Darmstadt, Germany) according to the manufacturer's protocol.

### 2.19 Detection of caspase activity

SEM cells were seeded onto 24‐well plate at a density of 0.2 × 10^6^ cells·mL^−1^ in their medium and exposed to AUR for 16 h. Detection of caspase activity (caspase‐1, caspase‐3, caspase‐4, caspase‐5, caspase‐6, caspase‐7, caspase‐8, and caspase‐9) was performed using Muse^®^ MultiCaspase Assay Kit (Merck Millipore) in accordance with the manufacturer's protocol and analyzed in Muse Cell Analyzer (Merck Millipore).

### 2.20 Data presentation and statistical analysis

Unless stated otherwise in Figure Legends, all the figures present averaged data from at least two independent experiments with standard errors of the mean (SEM). *P* values lower than 0.05 were considered statistically significant as measured by unpaired *t*‐test using graphpad7 Software (La Jolla, CA, USA) and presented as *P < 0.05, **P < 0.01, ***P < 0.0001. All other tests used for group comparisons are described in detail in Figure Legends.

## 3 Results and Discussion

### 3.1 BCP‐ALL cells have imbalanced redox homeostasis

**Figure 1:**
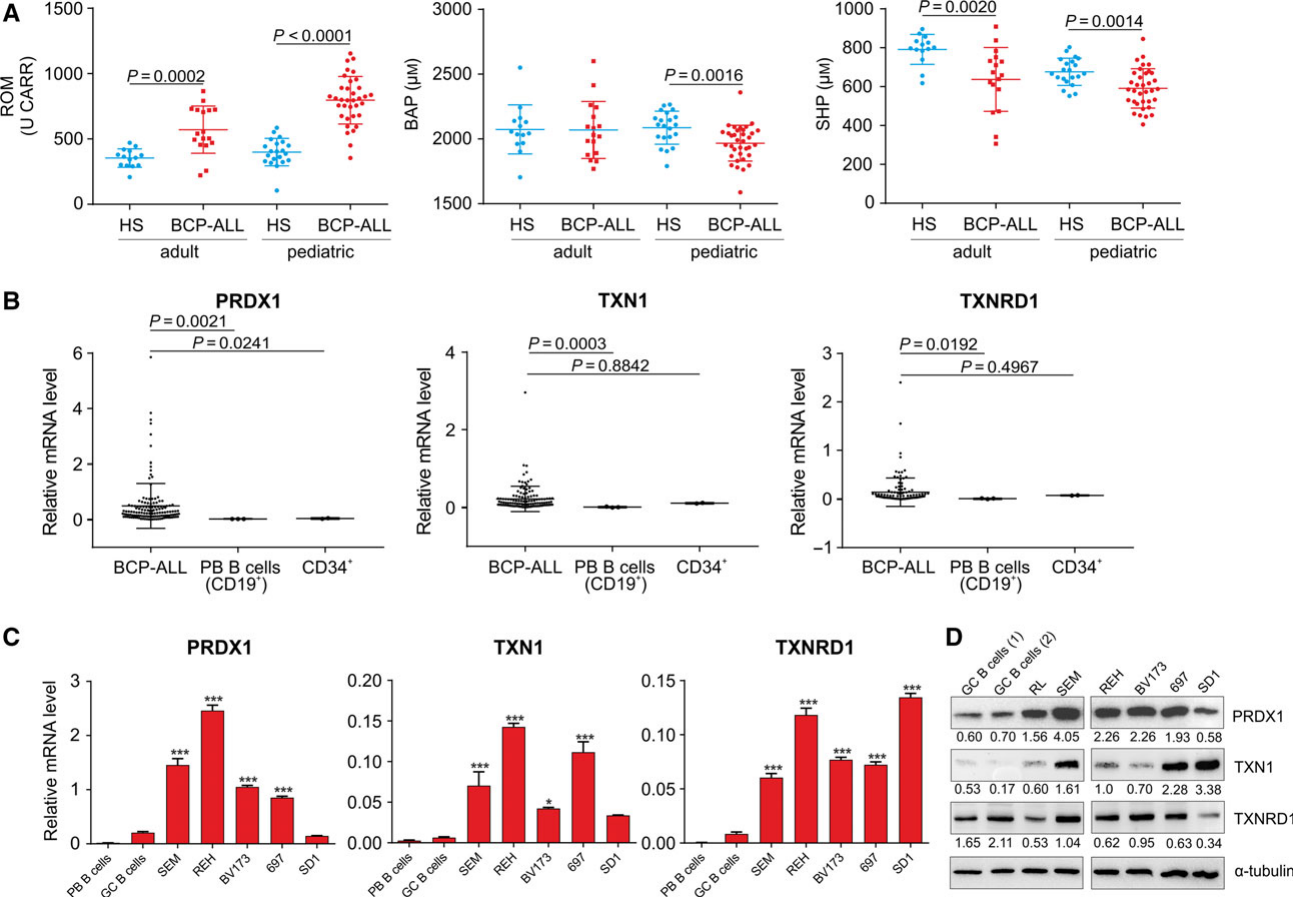
Increased oxidative stress in BCP‐ALL patients is accompanied by upregulation of TXN system antioxidant enzymes in BCP‐ALL primary cells and BCP‐ALL cell lines. (A) Redox biomarkers: ROM, BAP, and total thiol levels, as determined by free SH groups in proteins (SHP), were evaluated in the sera collected at diagnosis of BCP‐ALL pediatric (*n* = 35) and adult (*n* = 17) patients and compared with age‐related healthy subjects (HS, *n* = 21 and *n* = 14 for pediatric and adult HS, respectively). *P* values are presented for groups with significant differences (*P* < 0.05 in Mann–Whitney's *U*‐test). (B) mRNA levels of *PRDX1*,* TXN1*, and *TXNRD1* in primary BCP‐ALL blasts (in total *n* = 129 pediatric ALL for *PRDX1*,* n* = 115 pediatric ALL for *TXN1*,* TXNRD1*, and *n* = 11 adult ALL for all genes) and in normal CD34^+^ (total RNA from CD34^+^ cells isolated from peripheral blood of multiple donors, two independent samples) and normal CD19^+^ (isolated from peripheral blood of three donors) cells. mRNA expression of the TXN system antioxidant enzymes was assessed by qPCR normalized to a reference gene, 60S ribosomal protein L29 (*RPL29*), as $2^{-\Delta C_{t}}$. Data are presented as individual values with mean ± SD. *P* values for comparisons between BCP‐ALL patients’ groups and controls are presented (Mann–Whitney's *U*‐test). (C) mRNA levels of *PRDX1*,* TXN1*, and *TXNRD1* in BCP‐ALL cell lines were measured with qPCR and normalized to the housekeeping genes, *RPL29* and/or β2‐microglobulin (*B2M*). Data show mean values from at least two independent experiments + SEM and are compared with control peripheral blood (PB) B cells and tonsillar germinal center (GC) B cells isolated from two healthy donors. *P < 0.05, ***P < 0.0001 by ANOVA with Dunnett's *post hoc* test (compared with GC B cells). (D) Representative western blot for the protein levels of TXN antioxidant enzymes in malignant B‐cell lines and normal GC B cells. Relative intensity to α‐tubulin calculated by densitometry (image lab Software; Bio‐Rad Laboratories) is presented under each band.

To evaluate the redox homeostasis in BCP‐ALL patients, we measured the levels of redox status‐related parameters in BCP‐ALL patients’ sera collected at diagnosis. Reactive oxygen metabolites (ROM) were elevated in both adult and pediatric BCP‐ALL sera as compared to age‐related healthy subjects (Fig. 1A, left panel). In addition, antioxidant capacity was decreased in pediatric BCP‐ALL and free thiols were decreased in adult and pediatric BCP‐ALL sera, as measured by biological antioxidant potential (BAP) and the number of thiol groups in protein (SHP) assays, respectively (Fig. 1A, middle and right panels). None of the redox status‐related parameters positively correlated with white blood cell count (WBC) (ROM: *r* = 0.415 and −0.266, BAP: *r* = −0.046 and 0.095, SHP: *r* = −0.064 and −0.005, for pediatric and adult patients, respectively).

Next, we assessed the mRNA levels of the enzymes of the TXN system by qPCR in blasts isolated from patients diagnosed with BCP‐ALL. The mean mRNA level of *PRDX1* in BCP‐ALL blasts was higher compared to normal CD34^+^ or normal peripheral blood (PB) CD19^+^ cells, and the mean *TXN1* and *TXNRD1* levels were higher compared to normal PB CD19^+^ cells (Fig. 1B). The mRNA and protein levels of the TXN system enzymes were also determined in BCP‐ALL cell lines representing different cytogenetic subtypes of BCP‐ALL and compared to normal B cells isolated from peripheral blood (PB B cells) or from tonsillar germinal centers (GC B cells). At mRNA level, *TXNRD1* was upregulated in all cell lines, while *TXN1* and *PRDX1* were upregulated in the majority of cell lines, as compared to normal B cells (Fig. 1C). At the protein level, we observed significant upregulation of PRDX1 and TXN1 in a vast majority of cell lines (Fig. 1D).

Collectively, these data demonstrate disturbed redox homeostasis in BCP‐ALL patients’ sera, associated with increased levels of oxidative metabolites and reduced total antioxidant capacity and free protein thiols. These results are in agreement with previously published data showing elevated levels of other oxidative stress biomarkers (such as carbonylated proteins, malondialdehyde), and reduced levels of selected antioxidants in sera of BCP‐ALL patients (Battisti *et al*., 2008; Rasool *et al*., 2015), and substantiate the persistence of oxidative stress in BCP‐ALL patients. Furthermore, we observed that the dysregulation of redox homeostasis in BCP‐ALL sera is accompanied by increased expression of the TXN system antioxidant enzymes in BCP‐ALL cell lines and primary cells.

### 3.2 Inhibitors of the TXN antioxidant enzymes are effective against BCP‐ALL cell lines and primary cells *in vitro*

**Figure 2:**
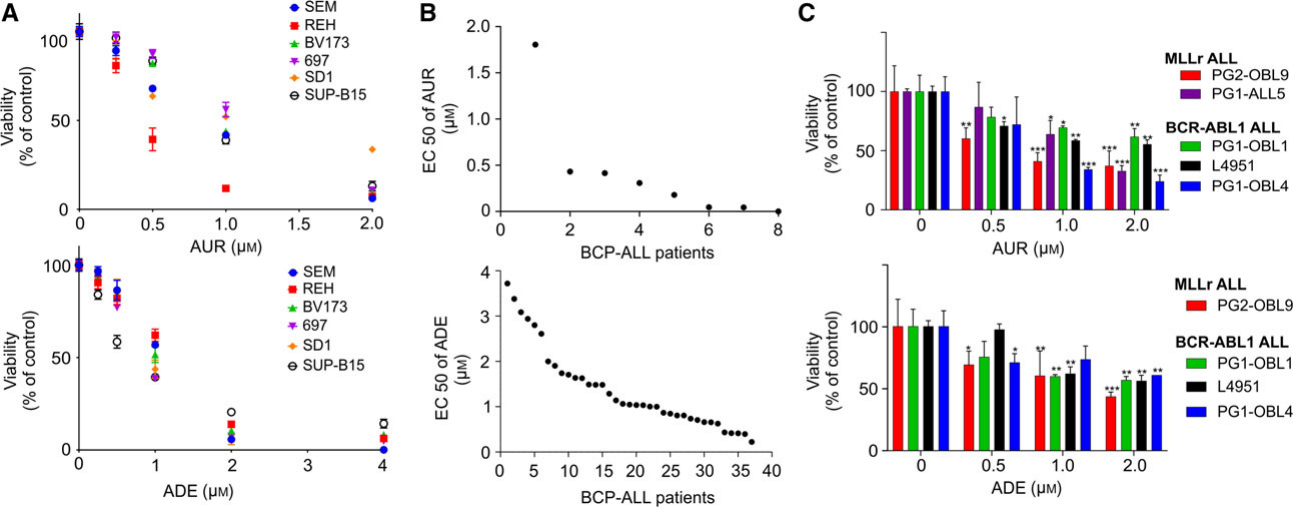
Inhibitors of the TXN system antioxidant enzymes effectively kill BCP‐ALL cell lines and primary blasts. (A) BCP‐ALL cell lines were treated with AUR and ADE at particular dose range (0.0625–2 μm for AUR and 0.125–4 μm for ADE) for 48 h and subjected to an MTT viability assay. Viability of the cells is shown as percentage of the untreated controls (DMSO). For each drug concentration, mean value is shown from at least two independent experiments ± SEM. (B) Adult BCP‐ALL cells (primary, *n* = 3 and primograft, *n* = 3) and pediatric primary cells (*n* = 2) were treated with AUR (0.0156–2 μm), whereas *n* = 37 pediatric primary cells were treated with ADE (0.25–8 μm) in monoculture for 4 days and subjected to MTT assay. Data are shown as EC_50_ of AUR and ADE, as calculated in graphpad7 (La Jolla, CA, USA) by nonlinear regression dose–response analysis. (C) Primograft cells representing MLLr (PG2‐OBL9, PG1‐ALL5) and BCR‐ABL1 (PG1‐OBL1, L4951, PG1‐OBL4) BCP‐ALL were cocultured with human primary BM‐MSC and incubated with indicated concentrations of AUR or ADE for 5 days (in two technical repeats for each drug concentration). Viability of ALL cells was assessed by a hemocytometer using trypan blue exclusion method. Number of the viable cells of each primograft is presented as percentage of the untreated controls (DMSO). For each primograft, the data are shown as means +SD. *P < 0.05, **P < 0.01, ***P < 0.0001 by ANOVA with Dunnett's *post hoc* test.

Considering the occurrence of oxidative stress in BCP‐ALL patients along with the upregulation of the antioxidant enzymes of the TXN system in BCP‐ALL cells, we hypothesized that in BCP‐ALL these enzymes may be components of the adaptation to the pro‐oxidant conditions. To address this hypothesis, we tested the cytostatic/cytotoxic efficacy of the TXNRD inhibitor auranofin (AUR) (Fiskus *et al*., 2014) (Fig. 2A, upper panel) as well as the PRDX1/2 and TXN inhibitor adenanthin (ADE) (Liu *et al*., 2012; Muchowicz *et al*., 2014) (Fig. 2A, lower panel) in BCP‐ALL cell lines. Both inhibitors affected the viability of all cell lines (Fig. 2A), with EC_50_ of 0.4–1.2 μm for AUR and 0.7–1.2 μm for ADE (Table S1). Moreover, AUR and ADE decreased the viability of primary BCP‐ALL blasts grown in monoculture, with EC_50_ in the range of 0.125–1.8 μm and 0.2–3.7 μm for AUR and ADE, respectively (Fig. 2B). Boutter *et al*. (2014) showed the role of bone marrow‐derived mesenchymal stem cells (BM‐MSC) in promoting survival and modulating of drug sensitivity of BCP‐ALL cells by an alleviation of oxidative stress. To test the cytostatic/cytotoxic efficacy of TXN system inhibitors in the presence of stroma, we employed an *in vitro* coculture model of BCP‐ALL cells with primary human BM‐MSC of confirmed phenotype and differentiation capacity (Fig. S1) (Pal *et al*., 2016). Despite stromal support and a redox‐protective cell culture medium supplemented with 2‐mercaptoethanol, we could observe cytotoxic effects of AUR and ADE to BCP‐ALL primografts harboring *MLL‐AF4* and *BCR‐ABL1* translocations (Fig. 2C). Next, we compared the cytotoxic efficacy (EC_50_) of AUR and ADE in BCP‐ALL blasts in monocultures and in corresponding cocultures with BM‐MSC. As presented in Table S2, in every case the presence of BM‐MSC increased resistance to both AUR and ADE.

These results demonstrate that both BCP‐ALL cell lines and primary cells are sensitive to low micromolar concentrations of AUR and ADE *in vitro*. The sensitivity is decreased but not eliminated when the BCP‐ALL cells are grown in a redox‐protective medium in the presence of primary BM‐MSC. Our observations align with the previously reported decreased cytotoxicity of AUR to a CLL cell line MEC‐1 grown in the presence of stromal, nurse‐like cells (Fiskus *et al*., 2014) and reveal the protective role of stromal cells in the sensitivity of BCP‐ALL cells to the TXN system inhibitors.

### 3.3 Auranofin delays leukemia progression in a patient‐derived xenograft model

Since the cytotoxic effect of plasma‐achievable concentrations of AUR to normal peripheral blood mononuclear cells (normal PBMC) was significantly weaker than ADE (Fig. S2A), we selected AUR for further studies. AUR exerted higher potency against malignant B cells, as compared to normal B cells (CD19^+^) and normal T cells (CD3^+^) (Fig. S2B). These observations are in agreement with previously published data reporting on selectivity of AUR to cancer cells (Fiskus *et al*., 2014; Stafford *et al*., 2018).

**Figure 3:**
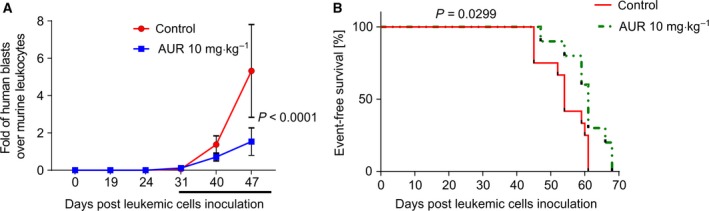
Auranofin attenuates BCP‐ALL progression and prolongs the survival of leukemic mice. (A) Primograft cells representing MLLr BCP‐ALL were injected into NSG mice, and leukemia progression was monitored weekly through peripheral blood staining with anti‐mCD45, anti‐hCD45, anti‐hCD19 antibodies. Starting from day 31 after the cell injection, randomly selected animals were treated with 10 mg·kg^−1^ of AUR or control (DMSO) through intraperitoneal injections, once a day for 3 weeks (5 days of treatment/2 days of break schedule, *n* = 7 for each group). Data are presented as a mean fold of human blasts (hCD45^+^/hCD19^+^) over murine leukocytes (mCD45^+^) ± SD in time from a representative experiment. Depicted *P* value was calculated by ANOVA with Bonferroni's correction. (B) Kaplan–Meier survival plot presenting event‐free survival of leukemic mice treated with AUR (10 mg·kg^−1^, *n* = 10) or vehicle (DMSO, *n* = 12) for 14 or 15 days. Data are pooled from two independent experiments. Depicted *P* value was calculated by log‐rank (Mantel–Cox) test.

Considering that AUR is a clinically used antirheumatic agent and is further tested for other indications including cancer (Roder and Thomson, 2015), we studied its efficacy against BCP‐ALL *in vivo* using a patient‐derived xenograft model. Since *in vitro* AUR was active against BCP‐ALL cells representing the MLL‐AF4 subtype derived from an adult patient, the subtype associated with extremely poor prognosis, we inoculated these cells to NSG mice. The mice were treated with AUR when the percentage of human blasts reached at least 1% murine leukocytes (1.4–23.9% at day 31 post‐leukemic cell inoculation), which indicates successful leukemia engraftment. AUR significantly delayed leukemia progression in mice during 3 weeks of the treatment and reduced the numbers of human blasts observed at the end of the treatment as compared to controls (Fig. 3A). Moreover, AUR prolonged the event‐free survival of leukemic mice (Fig. 3B). These data reveal that AUR attenuates the progression of MLLr BCP‐ALL *in vivo*.

### 3.4 Auranofin and adenanthin induce oxidative stress‐mediated cell death in BCP‐ALL cell lines and primary cells

**Figure 4:**
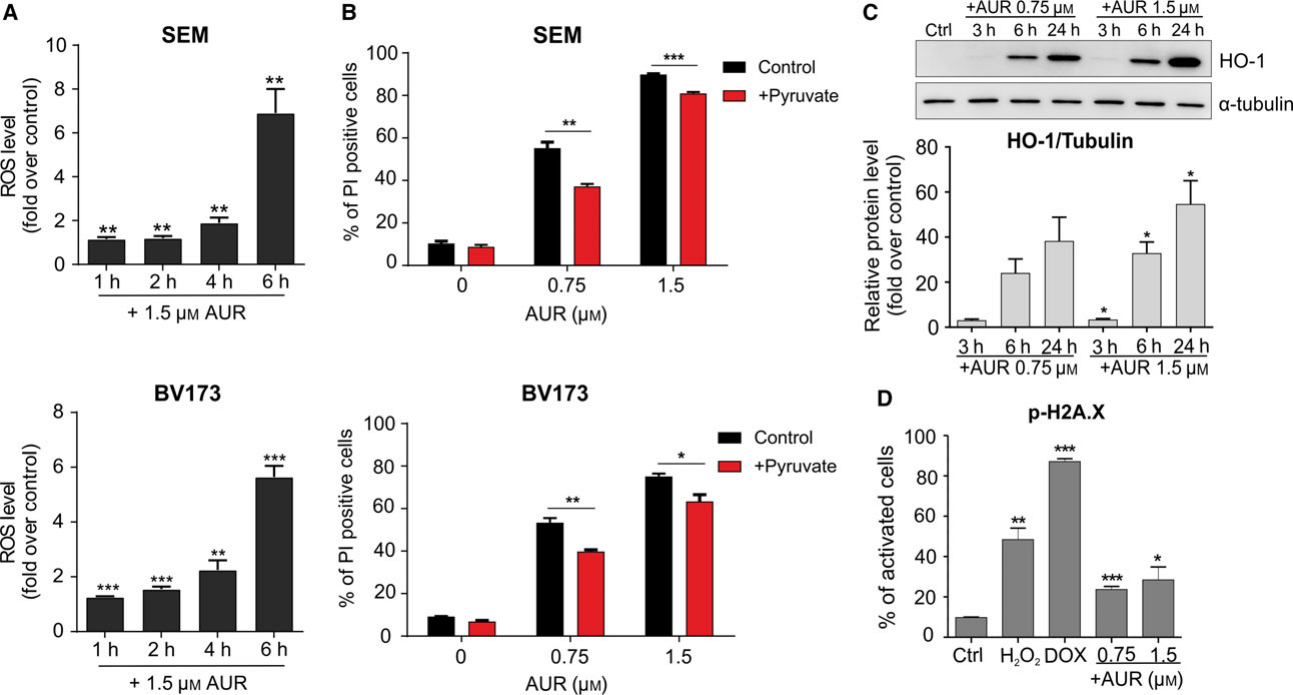
Inhibitors of the TXN system antioxidant enzymes trigger oxidative stress‐induced apoptosis in BCP‐ALL cell lines. (A) SEM and BV173 cells were exposed to 1.5 μm of AUR for 1, 2, 4, and 6 h, and ROS levels were accessed by CM‐H_2_‐DCFDA. Results are presented as fold change in mean fluorescence intensity (MFI) over untreated controls. The bars indicate mean + SEM from two independent experiments. (B) SEM and BV173 cells were pretreated with 1 mm of pyruvate for 30 min and then treated with EC_50_ and EC_80_ of AUR. After 24 h, the number of dead cells was assessed by flow cytometry after staining with propidium iodide (PI). The results are shown as mean percentage of PI‐positive cells + SEM from two independent experiments. (C) SEM cells were treated with EC_50_ and EC_80_ of AUR. The protein levels of the oxidative stress marker, heme oxygenase‐1 (HO‐1), were assessed by immunoblotting. Graph in the lower panel presents mean ratio of HO‐1 band intensity related to α‐tubulin band intensity, calculated by densitometry (image lab Software; Bio‐Rad Laboratories). The bars represent means + SEM from two independent experiments. (D) Phosphorylation of H2A.X, a marker of oxidative DNA damage, was measured using Muse^®^ Cell Analyzer (Merck Millipore). SEM cells were treated with EC_50_ and EC_80_ of AUR, whereas cells treated with 200 μm of H_2_O_2_ or 100 nm of doxorubicin (DOX) served as positive controls. After 24 h of incubation, cells were stained with anti‐phospho‐histone H2A.X (Ser139) and anti‐histone H2A.X antibodies and the percentages of activated cells (with phosphorylated histone H2A.X) were assessed in Muse Cell Analyzer. Data are shown as mean values + SEM from two independent experiments. *P < 0.05, **P < 0.01, ***P < 0.0001 by *t*‐test in A–D.

Next, we studied the mechanism of cell death triggered by AUR and ADE in two BCP‐ALL cell lines: SEM and BV173, representing two genetic subtypes of BCP‐ALL associated with poor prognosis: MLLr and BCR‐ABL1, respectively. AUR and ADE triggered an increase in ROS levels (Fig. 4A, Figs S3 and S4A,B), and 1 mm pyruvate, a hydrogen peroxide scavenger, partially rescued the cells from AUR and ADE cytotoxicity (Fig. 4B, Fig. S4C). Likewise, pretreatment of the cells with catalase, an enzyme which breaks down hydrogen peroxide, also reduced AUR cytotoxicity (Fig. S5). Accordingly, in SEM cells pyruvate or catalase fully abrogated AUR‐mediated ROS induction (Fig. S6). Moreover, both TXN system inhibitors induced the expression of heme oxygenase‐1 (HO‐1), a nuclear factor‐like‐2 (NRF2)‐regulated oxidative stress biomarker (Fig. 4C, Fig. S4D). ROS accumulation in cells augments the phosphorylation of histone 2A variant X (H2A.X), which is a marker of DNA damage (Li *et al*., 2006). Accordingly, in AUR/ADE‐treated SEM cells we observed a moderate increase in a phosphorylation of H2A.X (Ser139) (Fig. 4D, Fig. S7).

**Figure 5:**
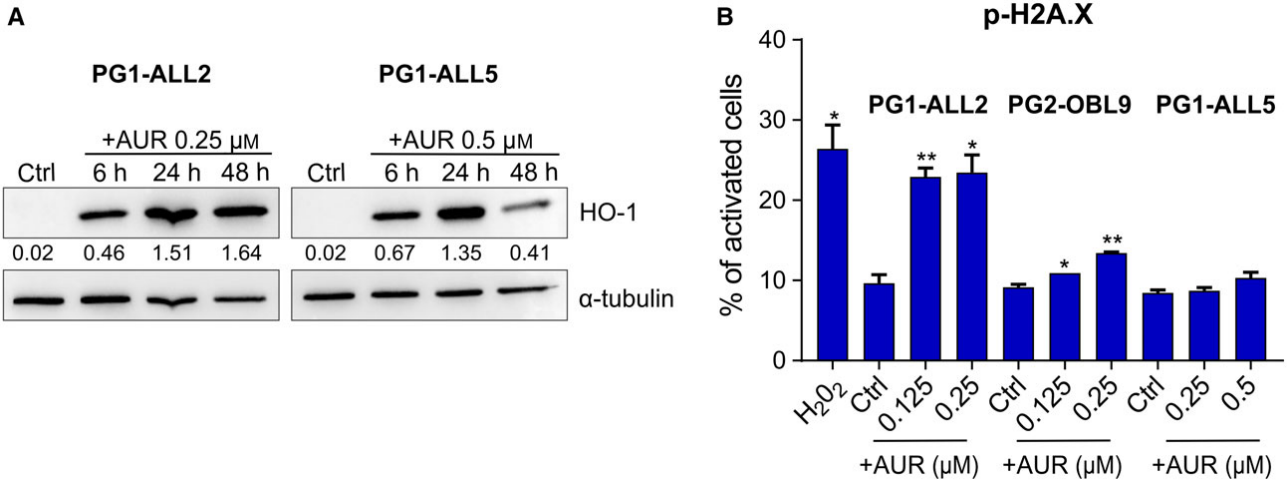
Auranofin upregulates oxidative stress‐related markers in BCP‐ALL primograft cells. (A) PG1‐ALL2 (BCR‐ABL1 BCP‐ALL) and PG1‐ALL5 (MLLr BCP‐ALL) primograft cells were exposed to indicated concentrations of AUR for 6, 24, and 48 h, and HO‐1 levels were evaluated by immunoblotting. Intensity ratio to α‐tubulin is presented under each band, calculated by densitometry. The experiment was performed once. (B) PG1‐ALL2, PG2‐OBL9 (MLLr BCP‐ALL), and PG1‐ALL5 primografts were exposed to AUR (concentrations between EC_50_ and EC_80_) or 200 μm of H_2_O_2_ (positive control performed in PG2‐OBL9 cells) for 24 h. Following the treatment, the phosphorylation of H2A.X was evaluated using Muse^®^ Cell Analyzer, as described in Fig. 4D and in Materials and methods. The bars represent mean values (two technical repeats) + SD (*n* = 1), *P < 0.05, **P < 0.01 by *t*‐test.

Further, we investigated the levels of oxidative stress biomarkers in response to AUR in primary BCP‐ALL cells. To this end, we treated primograft cells (representing MLLr and BCR‐ABL1 subtypes) with AUR. Similar to cell lines, in the primograft cells incubation with AUR induced the expression of HO‐1 and in two out of three primografts triggered the phosphorylation of H2A.X (Fig. 5A,B).

Altogether, the above results show that inhibitors of the TXN system trigger oxidative stress and a moderate cellular oxidative damage, both in BCP‐ALL cell lines and in primary cells. Furthermore, we present in BCP‐ALL cell line models that AUR triggers accumulation of hydrogen peroxide, which aligns with the hydrogen peroxide‐scavenging function of the TXN system (Arner, 2017). However, hydrogen peroxide only partially contributes to AUR‐mediated cell death, which may suggest that other mechanisms are also involved in cell death mediated by these inhibitors.

### 3.5 TXN system inhibitors induce ER stress and unfolded protein response

**Figure 6:**
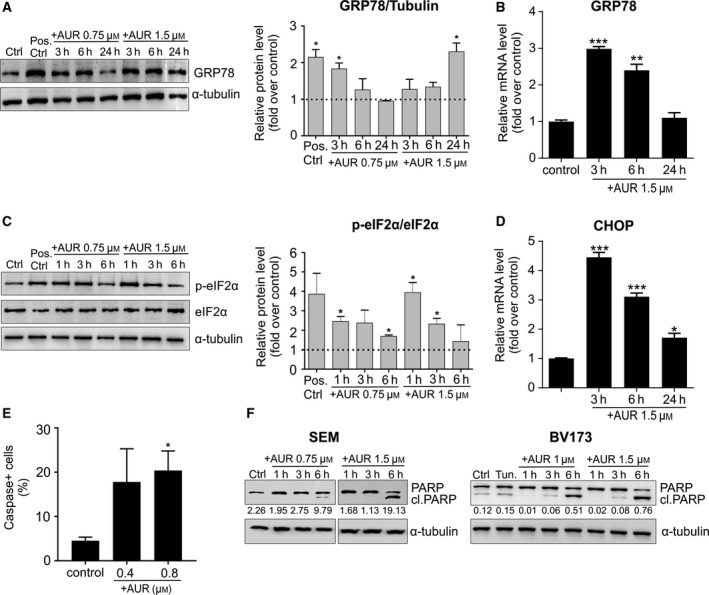
Inhibitors of the TXN system antioxidant enzymes trigger ER stress‐induced apoptosis. (A, B) For the detection of protein (western blotting, A) and mRNA (qPCR, B) levels of GRP78, SEM cells were treated with EC_50_ and/or EC_80_ of AUR and collected at indicated time points. Cells exposed to 10 μg·mL^−1^ of tunicamycin for 6 h served as a positive control. Densitometry analysis of western blot bands is shown as a mean intensity normalized to α‐tubulin and compared to controls. mRNA levels were detected by qPCR and normalized to a reference gene, *RPL29,* and presented as fold change over untreated controls. For both analyses, the bars represent means + SEM from two independent experiments. (C) To estimate eIF2α phosphorylation, SEM cells were incubated with indicated concentrations of AUR, lysed at three different time points, and analyzed by western blotting. Cells treated with 10 μg·mL^−1^ tunicamycin for 6 h served as a positive control. For western blot densitometry, p‐eIF2α mean intensity was normalized to eIF2α and compared to untreated controls. Each bar shows mean value +SEM from two independent experiments. (D) SEM cells were treated with EC_80_ of AUR, and mRNA levels of *CHOP* and control, *RPL29,* were measured by qPCR. Data are shown as fold change as compared to untreated controls. The bars indicate mean relative expressions + SEM from two independent experiments. (E) SEM cells were exposed to EC_20_ and EC_50_ of AUR for 16 h. Following the treatment, the cells were stained with fluorescent‐labeled inhibitor of caspases (FLICA), which allows detecting the cells with activated caspases using Muse Cell Analyzer. The data are presented as mean values + SEM from two independent repeats. (F) SEM and BV173 cells were incubated with EC_50_ and EC_80_ of AUR and collected after 1, 3, and 6 h. The graph shows representative blots for the protein levels of full‐length and cleaved PARP, and the intensity ratio of cleaved PARP to α‐tubulin is depicted under each band. *P < 0.05, **P < 0.01, ***P < 0.0001 by *t*‐test in A–E.

Auranofin has been shown to induce endoplasmic reticulum (ER) stress in CLL cells (Fiskus *et al*., 2014). Accordingly, we observed an induction of GRP78, a master regulator of ER stress‐induced unfolded protein response (UPR), at protein and mRNA levels already after 3 h of incubation with AUR and ADE (Fig. 6A,B, Fig. S8A,B). Additionally, AUR induced a phosphorylation of eIF2α (Fig. 6C), indicating the activation of a protein RNA‐like endoplasmic reticulum kinase (PERK) and a global translational block (Harding *et al*., 2000). We observed similar yet delayed and less pronounced effect in SEM cells treated with ADE (Fig. S8C).

The major goal of UPR is to provide a cytoprotective response to relieve ER stress, yet prolonged or intensified ER stress leads to apoptosis. C/EBP homologous protein (CHOP) is a major transcription factor linking ER stress with the induction of apoptosis (Zinszner *et al*., 1998). We observed a significant induction of *CHOP* expression in BCP‐ALL cells treated with AUR (Fig. 6D) and, to a lesser extent, also in cells treated with ADE (Fig. S8D). Accordingly, in BCP‐ALL cells treated with AUR we observed increased activity of caspases and poly(ADP‐ribose) polymerase (PARP) cleavage, indicating induction of caspase‐dependent apoptotic cell death (Fig. 6E,F).

These results show that inhibition of the TXN system in BCP‐ALL cell lines triggers PERK‐dependent, pro‐survival arms of the UPR pathways, such as induction of GRP78 expression and eIF2α phosphorylation, as well as induction of CHOP, an element of the switch from a cytoprotective to death‐promoting signaling. These results align with the previously reported induction of ER stress by AUR in CLL cells (Fiskus *et al*., 2014) and the recently suggested direct requirement of TXNRD1 for correct protein folding in the ER (Poet *et al*., 2017). Nonetheless, we do not exclude the possibility that other UPR‐related or UPR‐unrelated pathways may be involved in AUR‐mediated apoptosis. Therefore, further studies are required to better understand cell death signaling pathways accounting for AUR cytotoxicity in BCP‐ALL cells.

## 4 Conclusion

Collectively, our results highlight an important, pro‐survival role of the TXN system antioxidant enzymes in BCP‐ALL cells and the efficacy of their inhibitors, mainly AUR, as potential, novel drugs against BCP‐ALL. We report that increased oxidative stress biomarkers detected in BCP‐ALL patients’ sera are accompanied by an upregulation of antioxidant enzymes of the TXN system in BCP‐ALL cells, which may suggest that these enzymes promote survival under oxidative stress conditions. Accordingly, inhibitors of the TXN system antioxidant enzymes, AUR and ADE, effectively kill BCP‐ALL cell lines and BCP‐ALL primary cells as well as MLLr and BCR‐ABL1 primografts cocultured with BM‐MSC. We further show that AUR is more selective toward malignant B cells and it exerts cytotoxic effects to BCP‐ALL cells in concentrations that are possible to achieve in human plasma (Blocka *et al*., 1986). Moreover, AUR delays the progression of MLLr BCP‐ALL *in vivo* in patient‐derived xenograft model, further substantiating the efficacy of AUR to BCP‐ALL cells in the bone marrow niche. Similar to the previously published data obtained in other cancer models (Fiskus *et al*., 2014; Liu *et al*., 2012; Stafford *et al*., 2018), in BCP‐ALL cells AUR and ADE trigger accumulation of ROS, and induce oxidative macromolecule damage, UPR, and apoptosis. Although the induction of oxidative stress at least partially contributes to AUR‐mediated BCP‐ALL cell death, the involvement of UPR pathways in apoptotic signaling requires further investigations. Moreover, targets other than TXN system antioxidant enzymes, such as the previously reported deubiquitinases (Liu *et al*., 2014) or the glycolysis enzymes (Hou *et al*., 2018), could also contribute to the cytotoxic effect of AUR against BCP‐ALL cells. Altogether, this work unveils TXN system as a novel target for BCP‐ALL therapy and encourages further preclinical studies aimed at testing the efficacy of the combinations of TXN system inhibitors with anti‐BCP‐ALL chemotherapeutics, as well as with targeted drugs. Importantly, since AUR is a clinically approved drug, it could be easily repurposed in combination with existing drugs into therapy of BCP‐ALL.

## Conflict of interest

The authors declare no conflict of interest.

## Author contributions

KF performed, analyzed, and interpreted experiments, collected and assembled the data, and was involved in manuscript writing. AP performed, analyzed, and interpreted experiments, gathered patients’ material, and was involved in manuscript writing. WF analyzed and interpreted gene expression experiments, performed statistical analysis, and was involved in manuscript writing. AG, KSz, MAB, JM, JC, AG‐J, EJ, AM, AB, EP, ELM, EG‐M, UD, KG‐K, MM, and PP performed, analyzed, and interpreted experiments. DP, HB, and OH provided support and guidance in BCP‐ALL‐MSC coculture model, and participated in the experiment planning and data analysis. MW, PJ, WM, OH, and JG participated in experiment planning and analysis, and critically reviewed and edited the manuscript. MF conceived and designed the study, analyzed and interpreted the data, and wrote the manuscript. All authors have read and approved the final version of the manuscript.

## Supporting information


**Fig. S1.** BM‐MSC validation.
**Fig. S2.** The effects of AUR/ADE on normal and malignant cells.
**Fig. S3.** AUR induces ROS levels in SEM cell line.
**Fig. S4.** ADE increases oxidative stress in BCP‐ALL cell lines.
**Fig. S5.** Catalase partially reverses AUR‐induced cell death.
**Fig. S6.** Pyruvate and catalase prevent AUR‐mediated ROS induction.
**Fig. S7.** AUR and ADE induce DNA damage in SEM cell line.
**Fig. S8.** ADE induces ER stress.

**Table S1.** EC50 and EC80 of AUR and ADE for BCP‐ALL cell lines representing distinct subtypes of BCP‐ALL.
**Table S2.** EC50 of AUR and ADE for BCP‐ALL primograft cells treated in mono‐ and coculture with primary BM‐MSC.
**Table S3.** Clinical and biological characteristics of pediatric BCP‐ALL patients enrolled into TXN system genes expression analysis.
**Table S4.** Clinical and biological characteristics of adult BCP‐ALL patients enrolled into TXN system genes expression analysis.
**Table S5.** Sequences of primers used for qPCR.
**Table S6.** List of antibodies used for flow cytometry and immunoblotting.
**Table S7.** List of BCP‐ALL primografts used in *ex vivo* or/and *in vivo* studies.
