# Supplementary material for: Targeting the thioredoxin system as a novel strategy against B‐cell acute lymphoblastic leukemia
Source: Mol Oncol. 2019 Apr 5;13(5):1180–95. doi: 10.1002/1878-0261.12476 (PMC6487705; doi:10.1002/1878-0261.12476)
Supplement: Supplementary file 1 — Fig. S1. BM‐MSC validation. Fig. S2. The effects of AUR/ADE on normal and malignant cells. Fig. S3. AUR induces ROS levels in SEM cell line. Fig. S4. ADE increases oxidative stress in BCP‐ALL cell lines. Fig. S5. Catalase partially reverses AUR‐induced cell death. Fig. S6. Pyruvate and catalase prevent AUR‐mediated ROS induction. Fig. S7. AUR and ADE induce DNA damage in SEM cell line. Fig. S8. ADE induces ER stress. [file MOL2-13-1180-s001.doc]

**Supplementary Figures and Legends**


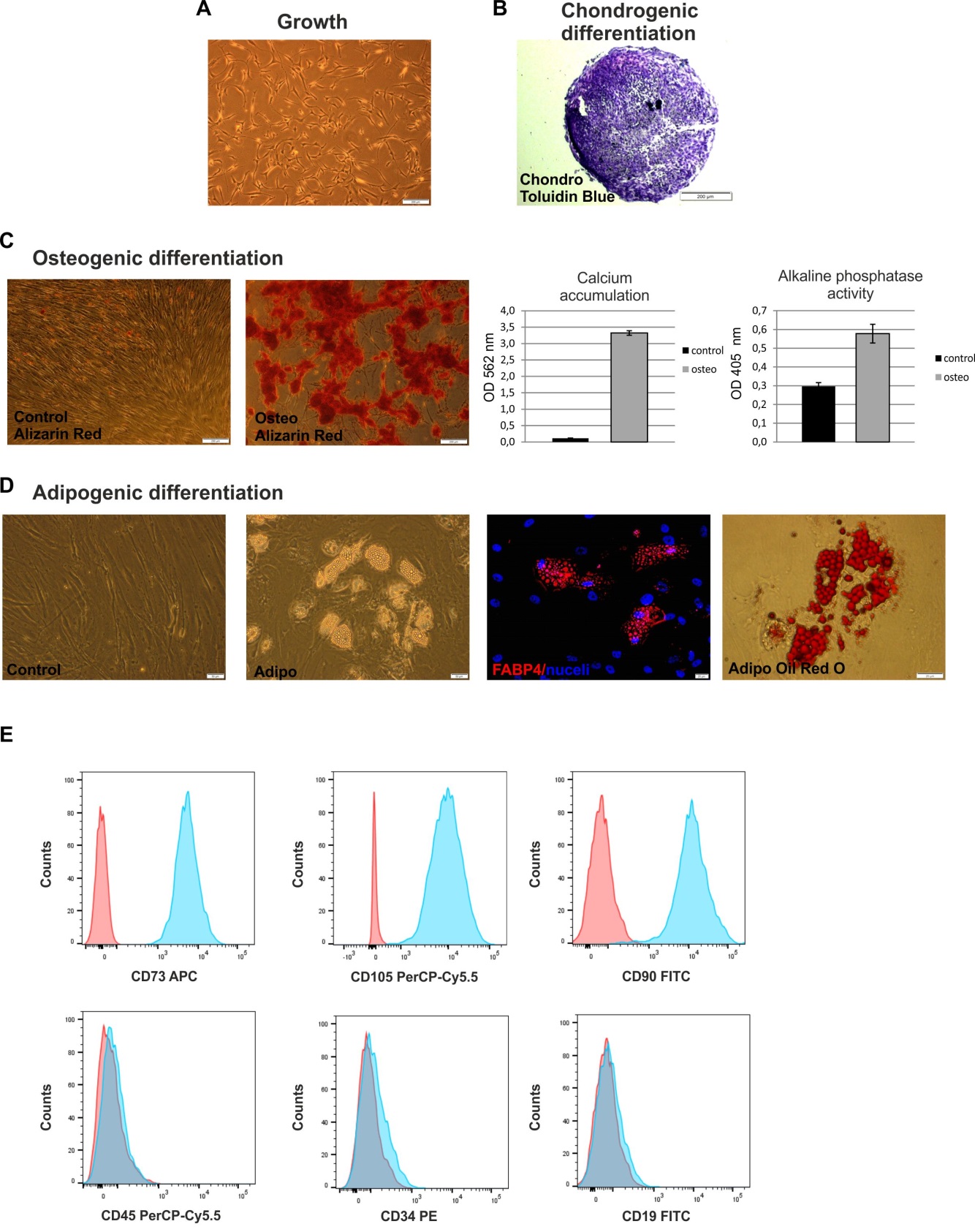


***Supplemental Fig. S1. BM-MSC validation.***

**A)** The morphology of undifferentiated BM-MSC. **B)** BM-MSC differentiated into chondropellet stained with toluidin blue. **C)** Osteogenic differentiation: the appearance in phase contrast microscope of undifferentiated BM-MSC (Control Alizarin Red) and after osteogenic differentiation stained with Alizarin Red (Osteo Alizarin Red); The graphs representing colorimetric analysis of calcium deposition (left graph) and alkaline phosphatase activity (right graph) in control and osteo-induced BM-MSC. **D)** Adipogenic differentiation: the appearance in phase contrast microscope of undifferentiated (Control) and following adipogenic differentiation (Adipo) BM-MSC; Immunocytochemistry – FABP4 stained in red (AF-594), cell nuclei stained in blue (DAPI); BM-MSC after adipogenic differentiation stained with Oil Red O; scale bars: A-C: 200 µm, D: 50 µm (Control, Adipo), 20 µm (FABP4/nuceli, Adipo Oil Red O). **E)** Immunophenotypic characterization of BM-MSC. Filled red histograms are BM-MSC unstained controls.


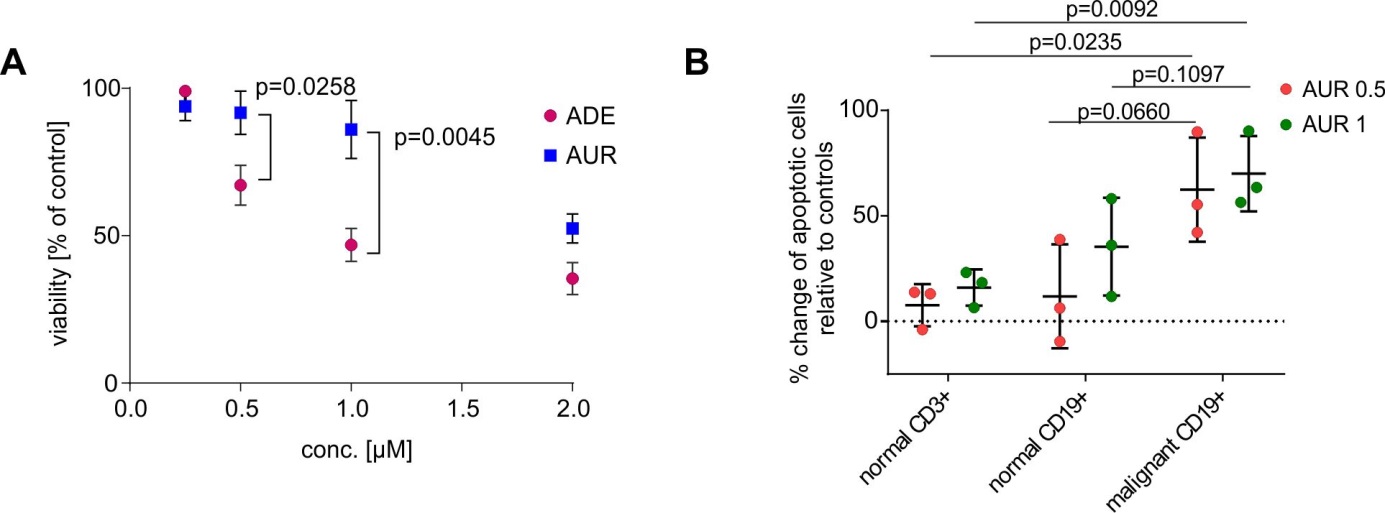


***Supplemental Fig. S2. The effects of AUR/ADE on normal and malignant cells.***

**A)** Normal PBMC were treated with AUR and ADE at indicated dose range for 4 days in monoculture and their viability was evaluated by MTT assay. Survival of the cells is shown as % of the untreated controls (DMSO). For each drug concentration, mean values are shown from 3 different donors ±SEM. Presented p values were calculated in *t*-test. **B)** PBMC isolated from blood of human healthy donors and primograft BCP-ALL cells isolated from spleens of NSG mice were treated with indicated concentrations of AUR for 4 days in monoculture. The number of dead CD3^+^ normal (n=3) and CD19^+^ normal (n=3) and CD19^+^ malignant (n=3) cells was assessed by flow cytometry after 7AAD staining. Results are presented as % change of apoptotic cells relative to controls, which was calculated according to the formula: [(% apoptotic cells in a group treated with AUR – % apoptotic cells in a control group)/% apoptotic cells in a group treated with AUR ]×100. Data present individual values with means ±SD. Presented p values were calculated in *t*-test.

***
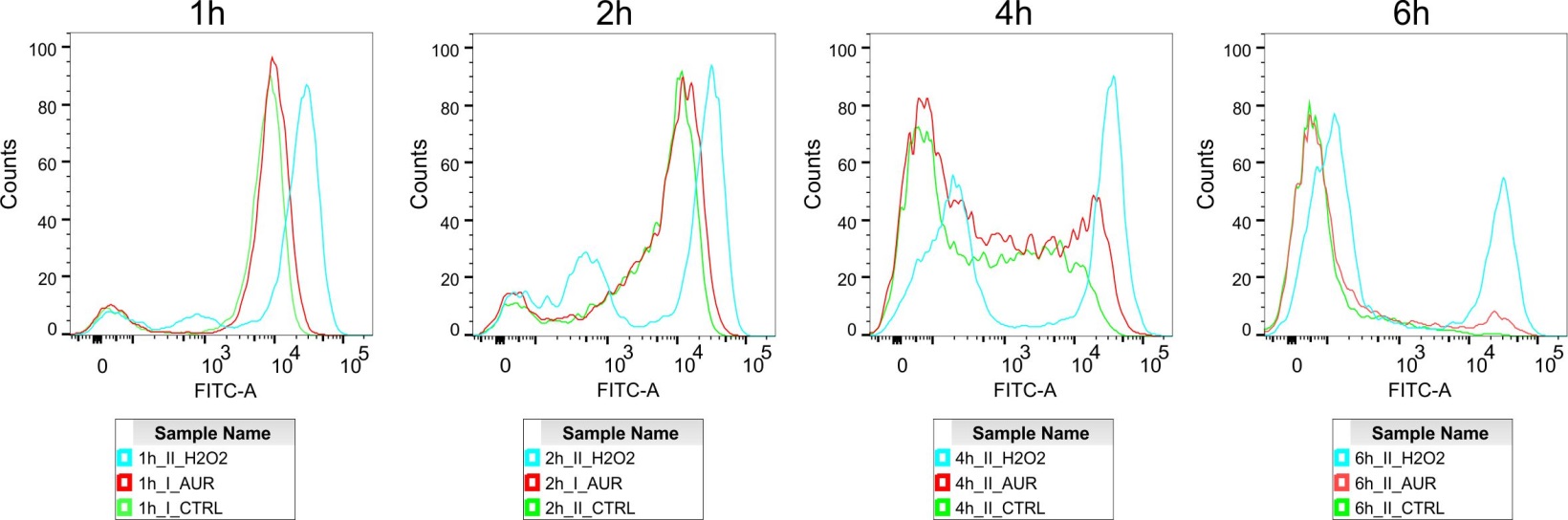
***

***Supplemental Fig. S3. AUR induces ROS levels in SEM cell line.***

Representative histograms showing green fluorescence intensity of CM-H2-DCFDA stained SEM cells subjected to 1.5 μM AUR and 100 µM H_2_O_2_ (positive control) at particular time-points.


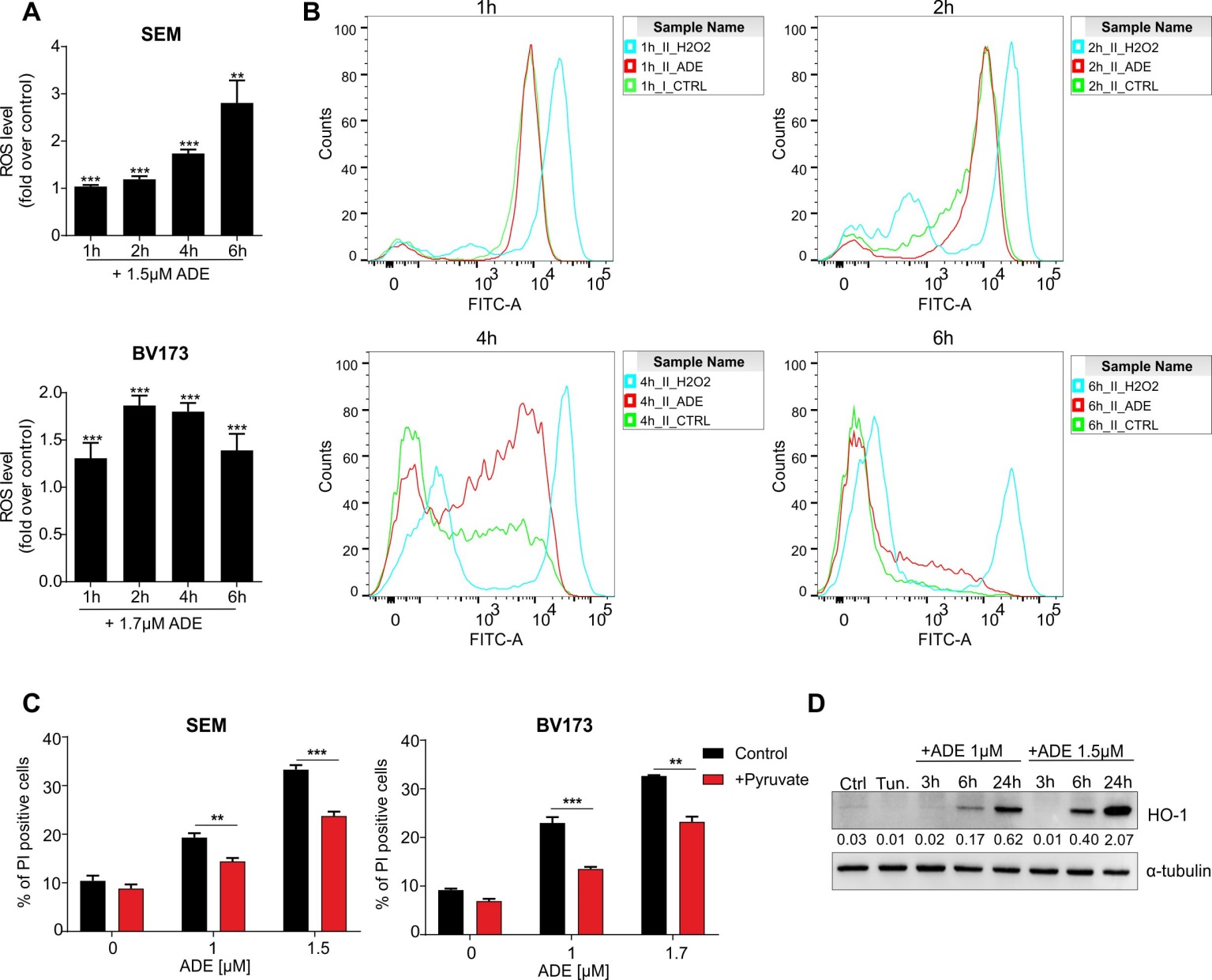


***Supplemental Fig. S4. ADE increases oxidative stress in BCP-ALL cell lines.***

**A)** SEM and BV173 cells were treated with 1.5 µM and 1.7 µM of ADE, respectively, for 1, 2, 4 and 6h. At each time-point the level of ROS was measured by CM-H2-DCFDA. Data are presented as fold change in mean fluorescence intensity (MFI) normalized to untreated controls. The bars show mean +SEM from 2 independent experiments. *P < 0.05, **P < 0.01, ***P < 0.0001 by t-test. **B)** Representative histograms showing green fluorescence intensity of CM-H2-DCFDA stained SEM cells treated with 1.5 μM ADE and 100 µM H_2_O_2_ (positive control) at particular time-points. **C)** SEM and BV173 cells were pre-incubated with 1mM of pyruvate for 30 min and subsequently treated with EC_50_ and EC_80_ of ADE calculated for each cell line. Following 24h incubation cells were stained with propidium iodide (PI) and % of dead cells were evaluated by flow cytometry. Bars represent mean +SEM (n=2). *P < 0.05, **P < 0.01, ***P < 0.0001 by t-test. **D)** SEM cells were exposed to EC_50_ and EC_80_ of ADE (calculated as described in *Fig. S1*) for indicated time and protein level of heme oxygenase 1 (HO-1) was evaluated by immunoblotting. Representative Western blot is shown and intensity ratio to α-tubulin is presented under each band.


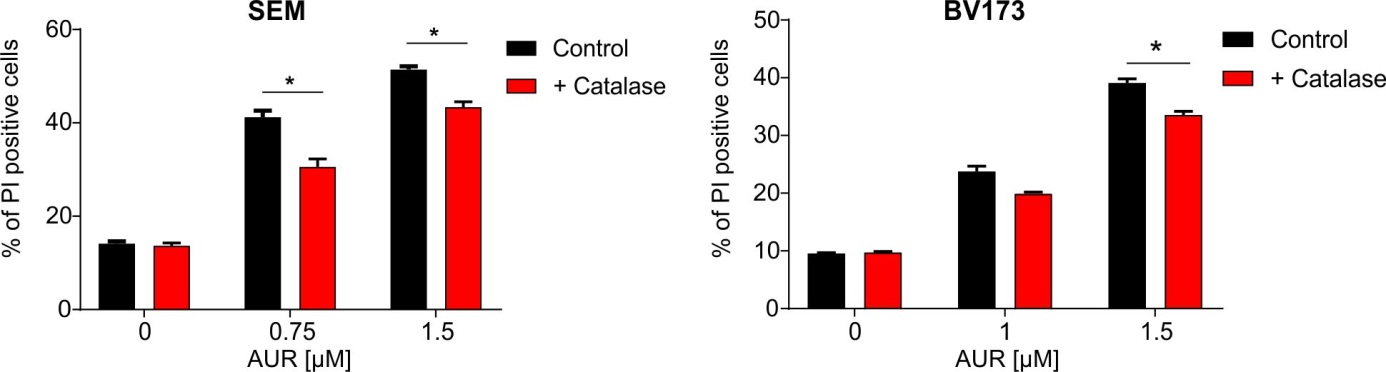


***Supplemental Fig. S5. Catalase partially reverses AUR-induced cell death.***

SEM and BV173 cells were pre-incubated with 100 µg/ml of catalase for 30 min and treated with EC_50_ and EC_80_ of AUR, calculated for each cell line. After 24h incubation, the percentage of dead cells was evaluated by flow cytometry using propidium iodide (PI) staining. Bars represent mean +SD (n=1). *P < 0.05 by t-test.


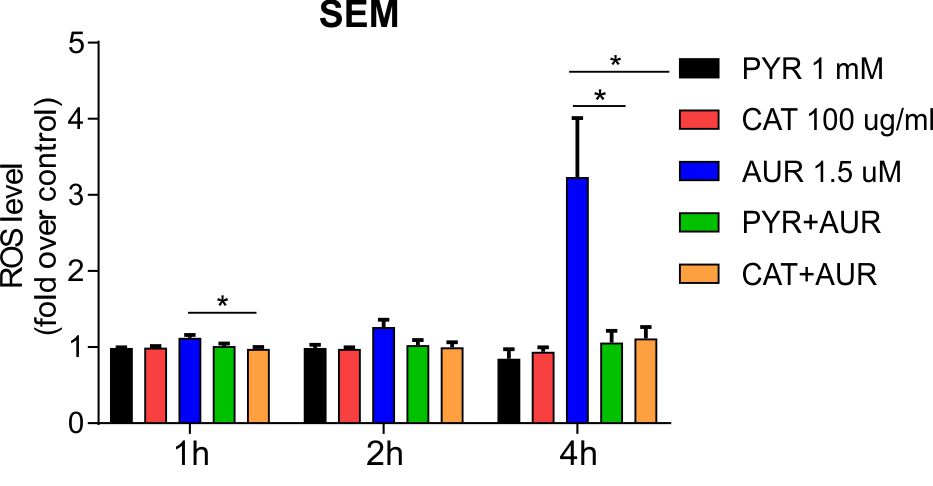


***Supplemental Fig. S6. Pyruvate and catalase prevent AUR-mediated ROS induction.***

SEM cells were pre-incubated with 1 mM of pyruvate or 100 µg/ml of catalase for 30 min and further treated with 1.5 µM of AUR for additional 1h, 2h and 4h. After each time-point the level of ROS was measured by CM-H2-DCFDA probe. Data are presented as fold change in mean fluorescence intensity (MFI) normalized to untreated controls. The bars show mean +SEM from 2 independent experiments. *P < 0.05, **P < 0.01, ***P < 0.0001 by t-test.


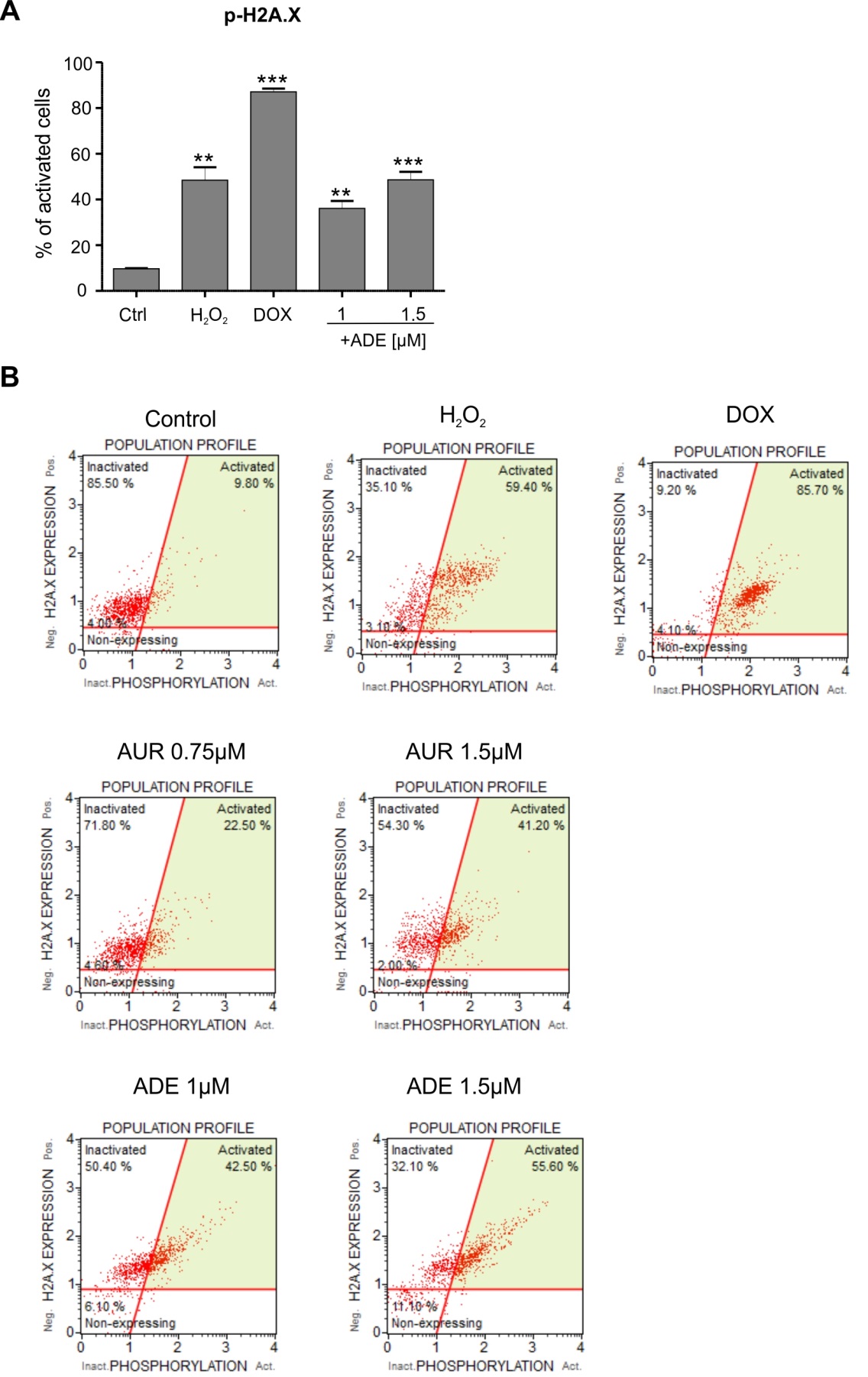


***Supplemental Fig. S7. AUR and ADE induce DNA damage in SEM cell line.***

Histone H2A.X phosphorylation was assessed using Muse® Cell Analyzer (Merck Millipore, Darmstadt, Germany). **A)** SEM cells were treated with EC_50_ and EC_80_ of ADE, while positive DNA damage controls with 200 μM of H_2_0_2_ or 100 nM of doxorubicin (DOX). Following 24h treatment cells were stained with anti-phospho-Histone H2A.X (Ser139) and an anti-Histone H2A.X and % of activated cells (with phosphorylated histone H2A.X) were evaluated. Bars show mean values +SEM from 2 independent experiments. *P < 0.05, **P < 0.01,
***P < 0.0001 by t-test. **B)** The results presented as dot-plots for SEM cells treated with EC_50_/EC_80_ of AUR and ADE and positive controls (H_2_0_2_, DOX). Upper quadrants present inactivated and activated cells expressing H2A.X. Activated cells (with H2A.X phosphorylation) indicate DNA damage.

*
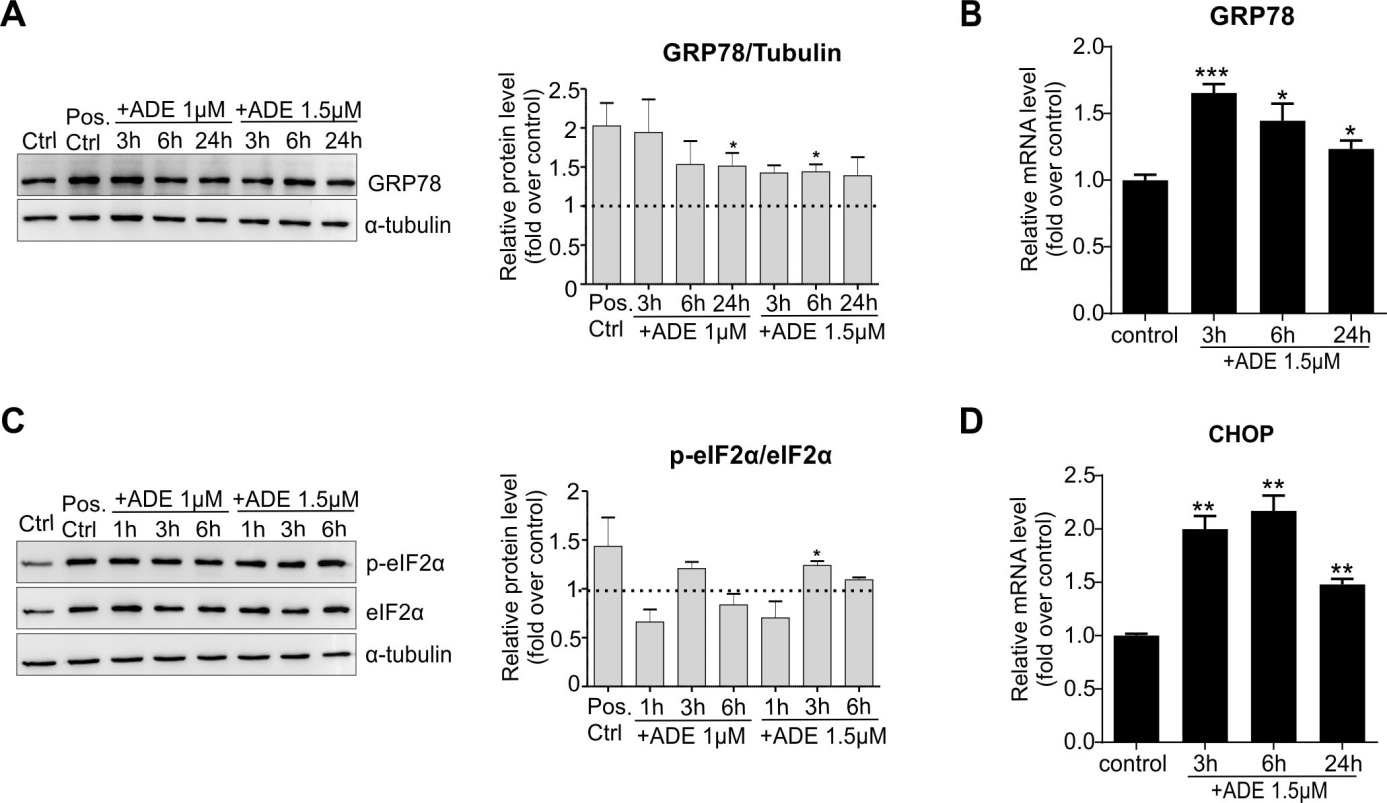
*

***Supplemental Fig. S8. ADE induces ER-stress.***

SEM cells were treated with EC_50_ and/or EC_80_ of ADE for 3, 6 and 24h. Cells incubated with 10 μg/ml of tunicamycin for 6h was taken as a positive control. Cells were collected after indicated time points for protein **A)** and mRNA **B)** levels of GRP78 measurement. Bands intensity is shown as a mean ratio normalized to α-tubulin and compared to untreated controls. mRNA levels were assessed by qPCR and normalized to housekeeping gene, *RPL29*. Data are shown as mean fold change as compared to controls +SEM from 2 independent experiments. **C)** SEM cells were incubated with indicated concentrations of ADE, lysed after 1, 3 and 6h and eIF2α phosphorylation was determined by immunoblotting. Cells exposed to 10 μg/ml of tunicamycin for 6h served as a positive control. For densitometry analysis, p-eIF2α to eIF2α ratio intensity was calculated and compared to untreated controls. Data are presented as a mean value +SEM from 2 independent repeats. **D)** SEM cells were exposed to 1.5 µM of ADE and mRNA level of *CHOP* was measured by qPCR. *CHOP* mRNA level is normalized to reference gene, *RPL29* and presented as fold change as compared to controls. Bars represent means +SEM for 2 independent experiments. *P < 0.05, **P < 0.01, ***P < 0.0001 by t-test in **A) – D).**
