# Supplementary material for: Targeting the thioredoxin system as a novel strategy against B‐cell acute lymphoblastic leukemia
Source: Mol Oncol. 2019 Apr 5;13(5):1180–95. doi: 10.1002/1878-0261.12476 (PMC6487705; doi:10.1002/1878-0261.12476)
Supplement: Supplementary file 2 — Table S1. EC50 and EC80 of AUR and ADE for BCP‐ALL cell lines representing distinct subtypes of BCP‐ALL. Table S2. EC50 of AUR and ADE for BCP‐ALL primograft cells treated in mono‐ and coculture with primary BM‐MSC. Table S3. Clinical and biological characteristics of pediatric BCP‐ALL patients enrolled into TXN system genes expression analysis. Table S4. Clinical and biological characteristics of adult BCP‐ALL patients enrolled into TXN system genes expression analysis. Table S5. Sequences of primers used for qPCR. Table S6. List of antibodies used for flow cytometry and immunoblotting. Table S7. List of BCP‐ALL primografts used in ex vivo or/and in vivo studies. [file MOL2-13-1180-s002.doc]

**Supporting Information**

# Targeting the thioredoxin system as a novel strategy against B cell acute lymphoblastic leukemia

Klaudyna Fidyt^1,2^, Agata Pastorczak^3^, Agnieszka Goral^1^, Kacper Szczygiel^1^, Wojciech Fendler^4,5^, Angelika Muchowicz^1^, Marcin Adam Bartlomiejczyk^3^, Joanna Madzio^2,3^, Julia Cyran^1^, Agnieszka Graczyk-Jarzynka^1^, Eugene Jansen^6^, Elzbieta Patkowska^7^, Ewa Lech-Maranda^7,8^, Deepali Pal^9^, Helen Blair^9^, Anna Burdzinska^10^, Piotr Pedzisz^11^, Eliza Glodkowska-Mrowka^12^, Urszula Demkow^12^, Karolina Gawle-Krawczyk^13^, Michal Matysiak^13^, Magdalena Winiarska^1^, Przemyslaw Juszczynski^7^, Wojciech Mlynarski^3^, Olaf Heidenreich^9^, Jakub Golab^1,14,#^, Malgorzata Firczuk^1,#^

^1^Department of Immunology, Medical University of Warsaw, Warsaw, Poland

^2^Postgraduate School of Molecular Medicine, Medical University of Warsaw, Warsaw, Poland

^3^Department of Pediatrics, Oncology, Hematology and Diabetology, Medical University of Lodz, Lodz, Poland

^4^Department of Biostatistics and Translational Medicine, Medical University of Lodz, Lodz, Poland

^5^Department of Radiation Oncology, Dana-Farber Cancer Institute, Boston, MA, USA

^6^Centre for Health Protection, National Institute for Public Health and the Environment, Bilthoven, the Netherlands

^7^Institute of Hematology and Transfusion Medicine, Warsaw, Poland

^8^Centre of Postgraduate Medical Education, Warsaw, Poland

^9^Newcastle Cancer Centre at the Northern Institute for Cancer Research, Newcastle University, Newcastle upon Tyne, United Kingdom

^10^Department of Immunology, Transplantology and Internal Diseases, Medical University of Warsaw, Warsaw, Poland

^11^Department of Orthopaedics and Traumatology, Medical University of Warsaw, Warsaw, Poland

^12^Department of Laboratory Diagnostics and Clinical Immunology of Developmental Age, Medical University of Warsaw, Warsaw, Poland

^13^Department of Pediatrics, Hematology and Oncology, Medical University of Warsaw, Warsaw, Poland

^14^Centre for Preclinical Research and Technology, Medical University of Warsaw, Warsaw, Poland

^#^corresponding authors: Malgorzata Firczuk, email: [mfirczuk@wum.edu.pl](mailto:mfirczuk@wum.edu.pl) and Jakub Golab, email: [jakub.golab@wum.edu.pl](mailto:jakub.golab@wum.edu.pl), Department of Immunology, Medical University of Warsaw, Nielubowicza 5 Street, 02-097 Warsaw, Poland

**Supplemental Table 1.**

**EC50 and EC80 of AUR and ADE for BCP-ALL cell lines representing distinct subtypes of BCP-ALL.** Based on MTT results, EC values were calculated by nonlinear regression dose-response analysis using GraphPad7. For each cell line, EC50 and EC80 are presented as a mean ±SD from at least 2 independent repeats.

**
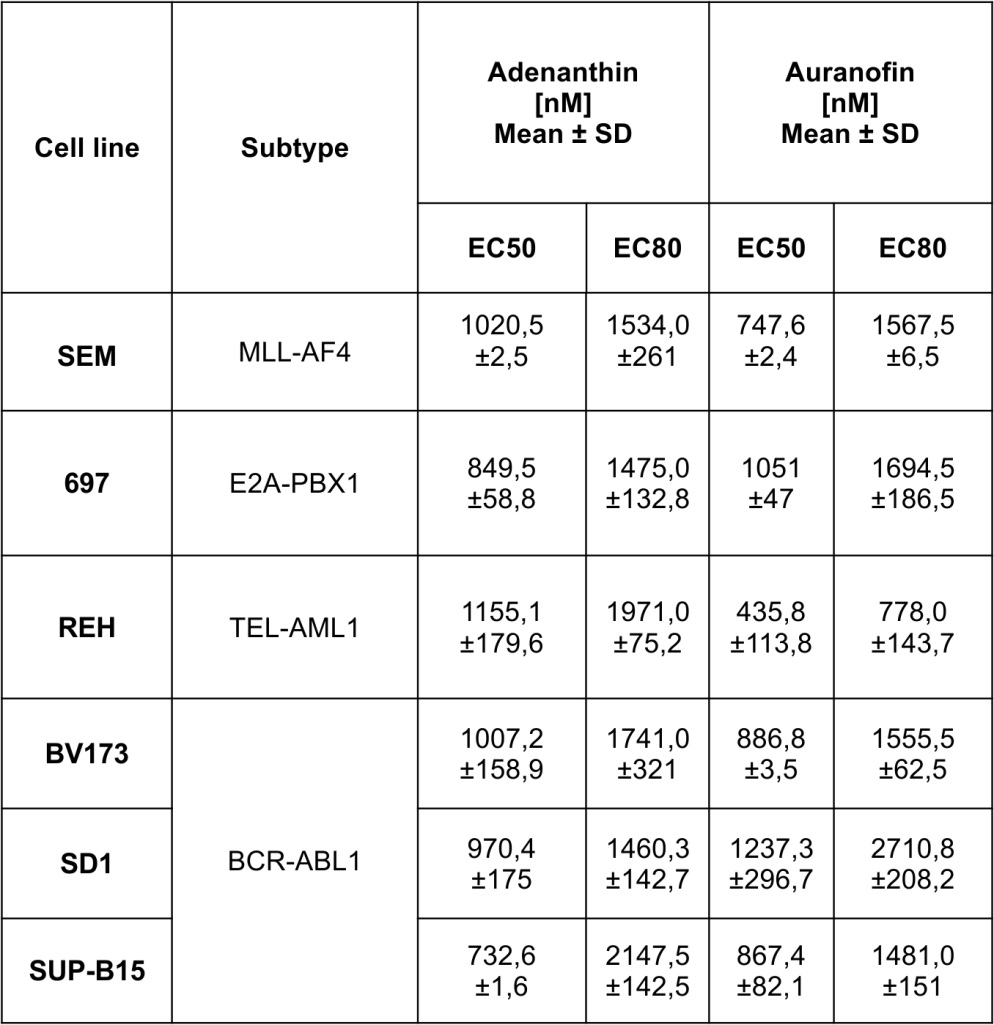
**

**Supplemental Table 2. EC50 of AUR and ADE for BCP-ALL primograft cells treated in mono- and co-culture with primary bone marrow derived mesenchymal stem cells (BM-MSC).** Based on MTT results (mono-culture) and trypan blue exclusion (co-culture), EC values were calculated by nonlinear regression dose-response analysis using GraphPad7.


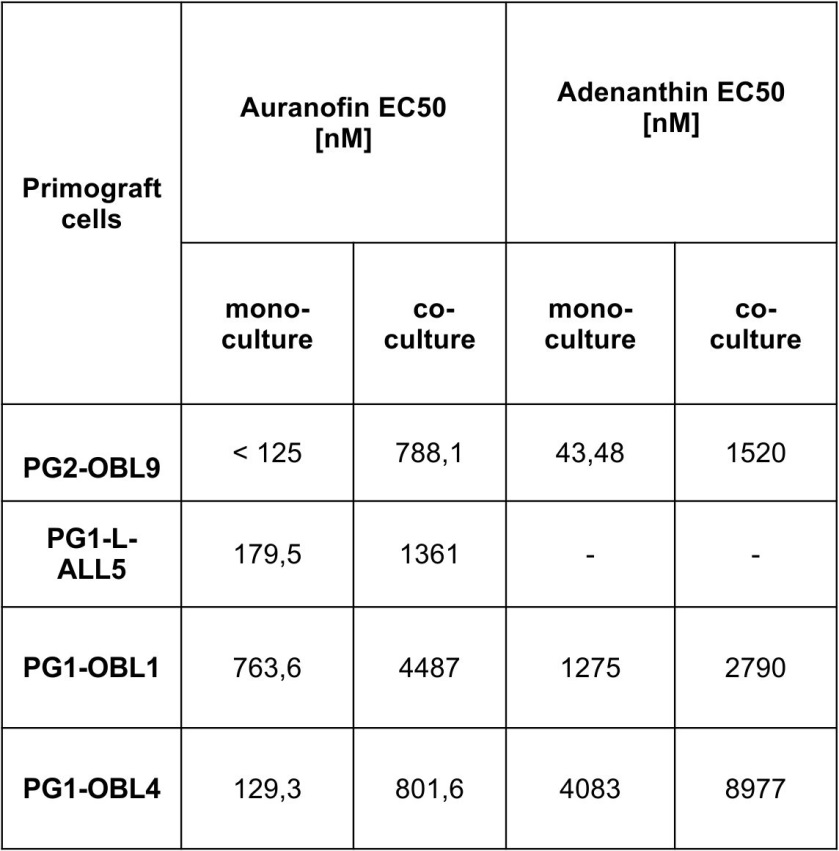


**Supplemental Table 3. Clinical and biological characteristics of pediatric BCP-ALL patients enrolled into TXN system genes expression analysis.**

| **Variable** | **Pediatric BCP-ALL**  **n=129** |
| --- | --- |
| **Median age (years)**^b^ | 4.5 [2.82-8.07] |
| **Males**^a^ | 60 (46.5) |
| **WBC count (×10^3^/mm^3^)**^b^ | 16.53 [4.9-63.7] |
| **Poor steroid response**^a^ | 13 (10) |
| **Hipodiploidy**^a^ | 0 (0.0) |
| ***MLL* rearrangements**^a^ | 14 (10.8) |
| ***ETV6-RUNX1* fusion**^a^ | 16 (12.4) |
| ***BCR-ABL1* fusion**^a^ | 6 (4.6) |
| **Median MRD15 (%)**^b^ | 0.38 [0.04-3.00] |
|  |  |

^a^ Shown as number of cases, with percentages in brackets.

^b^ Shown as median value with interquartile range shown in square brackets.
MRD15 – minimal residual disease evaluated at day 15.

**Supplemental Table 4. Clinical and biological characteristics of adult BCP-ALL patients enrolled into TXN system genes expression analysis.**

| **Variable** | **Adult BCP-ALL**  **n=11** |
| --- | --- |
| **Median age (years)^b^** | 55 [42-68] |
| **Males^a^** | 6 (55) |
| **WBC count (×10^3^/mm^3^)^b^** | 64.91 [6.9-163.3] |
| **Hipodiploidy^a^** | 2 (18.2) |
| ***MLL* rearrangements^a^** | 1 (9.1) |
| ***ETV6-RUNX1* fusion^a^** | 0 (0.0) |
| ***BCR-ABL1* fusion^a^** | 4 (36.4) |
| **Median MRD34-35 (%)^b^** | 0.00 [0.00-0.425] |

^a^ Shown as number of cases, with percentages in brackets.

^b^ Shown as median value with interquartile range shown in square brackets.

MRD34-35 – minimal residual disease evaluated at day 34-35.

**Supplemental Table 5. Sequences of primers used for qPCR.**

| **Primers** | **Sequences** |
| --- | --- |
| PRDX1_F | CACTGACAAACATGGGGAAGT |
| PRDX1_R | TTTGCTCTTTTGGACATCAGG |
| PRDX2_F | GCCTTCCAGTACACAGACGAG |
| PRDX2_R | GTTGGGCTTAATCGTGTCACT |
| TXN1_F | TAAAGGGAGAGAGCAAGCAG |
| TXN1_R | CAGAGAGGGAATGAAAGAAAGG |
| TXNRD1_F | TCACCCCAGTTGCAATCC |
| TXNRD1_R | GGTTGGAACATTTTCATAGTCACA |
| TXNIP_F | GGCGGGTGTCTGTCTCTGCT |
| TXNIP_R | GGCAAGGTAAGTGTGGCGGG |
| CHOP_F | AGCTCTGATTGACCGAATGG |
| CHOP_R | GATTGAGGGTCACATCATTGG |
| GRP78_F | AGCAGGACATCAACTCTTGC |
| GRP78_R | CCTTCTTTCCCAAATAAGCCT |
| B2M_F | TAGGAGGGCTGGCAACTTAG |
| B2M_R | CCAAGATGTTGATGTTGGATAAGA |
| RPL_F | CAGCTCAGGCTCCCAAAC |
| RPL_R | GCACCAGTCCTTCTGTCCTC |

**Supplemental Table 6. List of antibodies used for flow cytometry and immunoblotting.**

| **Antibody** | **Clone** | **Company** |
| --- | --- | --- |
| **Antibodies for flow cytometry** | | |
| anti-CD73, CD105, CD90 - BD Stemflow™ hMSC Analysis Kit | AD2, 266, 5E10 | BD Biosciences, Franklin Lakes, NJ, USA |
| anti-mCD45 APC | 30-F11 | eBioscience, San Diego, CA, USA |
| anti-hCD45 PE | HI30 | eBioscience, San Diego, CA, USA |
| anti-hCD34 APC | 4H11 | eBioscience, San Diego, CA, USA |
| anti-hCD19 FITC | HIB19 | eBioscience, San Diego, CA, USA |
| anti-hCD19 APC | HIB19 | BD Biosciences, Franklin Lakes, NJ, USA |
| anti-hCD3 PE-Cy7 | OKT3 | eBioscience, San Diego, CA, USA |
| **Antibodies for immunoblotting** | | |
| anti-PRDX1 | polyclonal | Sigma-Aldrich, St. Louis, USA |
| anti-PRDX2 | EPR5154 | GeneTex Inc., Irvine, CA, USA |
| anti-TXN1 | C63C6 | Cell Signaling, Beverly, MA, USA |
| anti-TXNRD1 | 19A1 | Abcam, Cambridge, UK |
| anti-HO-1 | polyclonal | Cell Signaling, Beverly, MA, USA |
| anti-GRP78 | polyclonal | Cell Signaling, Beverly, MA, USA |
| anti-p-eIF2α (Ser51) | D9G8 | Cell Signaling, Beverly, MA, USA |
| anti-eIF2α | L57A5 | Cell Signaling, Beverly, MA, USA |
| anti-PARP | polyclonal | Cell Signaling, Beverly, MA, USA |
| anti-α-tubulin | DM1A | Calbiochem, San Diego, CA, USA |

**Supplemental Table 7. List of primograft BCP-ALL used in *ex vivo* or/and *in vivo* studies.**

| **Primograft sample** | **BCP-ALL subtype** | **Age at diagnosis** |
| --- | --- | --- |
| PG1-OBL9 | MLL-AF4 | 48 years |
| PG1-ALL5 | MLL-AF4 | 11 years |
| L4951 | BCR-ABL1 | 15 years |
| PG1-ALL2 | BCR-ABL1 | 16 years |
| PG1-OBL1 | BCR-ABL1 | 55 years |
| PG1-OBL4 | BCR-ABL1 | 41 years |
